# SLC26A9 deficiency causes gastric intraepithelial neoplasia in mice and aggressive gastric cancer in humans

**DOI:** 10.1007/s13402-022-00672-x

**Published:** 2022-04-14

**Authors:** Xuemei Liu, Taolang Li, Zhiyuan Ma, Brigitte Riederer, Dumin Yuan, Jiaxing Zhu, Yunhua Li, Jiaxing An, Guorong Wen, Hai Jin, Xiao Yang, Ursula Seidler, Biguang Tuo

**Affiliations:** 1 Department of Gastroenterology, Affiliated Hospital of Zunyi Medical University, Dalian Road 149, Zunyi, 563000 China; 2 Department of General Surgery, Affiliated Hospital of Zunyi Medical University, Dalian Road 149, Zunyi, 563000 China; 3 Department of Gastroenterology, Hepatology and Endocrinology, Hannover Medical School, Carl-Neuberg-Straße 1, 30625 Hannover, Germany; 4 State Key Laboratory of Proteomics, Beijing Proteome Research Center, National Center for Protein Sciences (Beijing), Beijing Institute of Lifeomics, Beijing, 102206 China

**Keywords:** Gastric carcinogenesis, Slc26a9, Gastric parietal cells, Wnt signaling

## Abstract

**Background:**

Solute carrier family 26 member (SLC26A9) is a Cl^−^ uniporter with very high expression levels in the gastric mucosa. Here, we describe morphological and molecular alterations in gastric mucosa of *slc26a9*^−/−^ mice and in selective parietal cell-deleted *slc26a9*^*fl/fl*^*/Atp4b-Cre* mice and correlate SLC26A9 expression levels with morphological and clinical parameters in a cohort of gastric cancer (GC) patients.

**Methods:**

The expression patterns of genes related to transport and enzymatic function, proliferation, apoptosis, inflammation, barrier integrity, metaplasia and neoplasia development were studied by immunohistochemistry (IHC), quantitative RT-PCR, in situ hybridization and RNA microarray analysis. SLC26A9 expression and cellular/clinical phenotypes were studied in primary human GC tissues and GC cell lines.

**Results:**

We found that both complete and parietal cell-selective Slc26a9 deletion in mice caused spontaneous development of gastric premalignant and malignant lesions. Dysregulated differentiation of gastric stem cells in an inflammatory environment, activated Wnt signaling, cellular hyperproliferation, apoptosis inhibition and metaplasia were observed. Analysis of human gastric precancerous and cancerous tissues revealed that SLC26A9 expression progressively decreased from atrophic gastritis to GC, and that downregulation of SLC26A9 was correlated with patient survival. Exogenous expression of SLC26A9 in GC cells induced upregulation of the Cl^−^/HCO_3_^−^ exchanger AE2, G2/M cell cycle arrest and apoptosis and suppressed their proliferation, migration and invasion.

**Conclusions:**

Our data indicate that SLC26A9 deletion in parietal cells is sufficient to trigger gastric metaplasia and the development of neoplastic lesions. In addition, we found that SLC26A9 expression decreases during human gastric carcinogenesis, and that exogenous SLC26A9 expression in GC cells reduces their malignant behavior.

**Graphical abstract:**

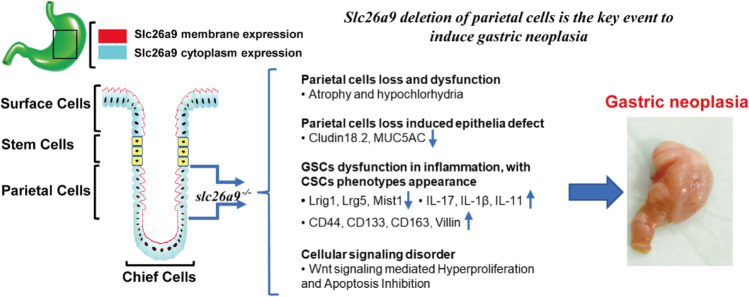

**Supplementary Information:**

The online version contains supplementary material available at 10.1007/s13402-022-00672-x.

## Introduction

Gastric cancer (GC) is a common malignant tumor and a major health threat worldwide. Gastric carcinogenesis is believed to result from interactions between *Helicobacter pylori (H. pylori)* infection and genetic, epigenetic and environmental factors in a multistep process of histological progression from atrophic gastritis (AG) through intestinal metaplasia (IM) to GC [1, 2]. In this classic “Correa sequence”, AG with parietal cell loss is a critical initial step in GC development [1, 2].

Solute carrier family 26 member 9 (Slc26a9) is a member of the Slc26a family of multifunctional anion transporters [3] and has recently been characterized as a Cl^−^ uniporter that is strongly expressed in gastric surfaces and glandular epithelia in both mice and humans [4–7]. Previously, it has been found that conditional loss of Slc26a9 expression in the murine stomach results in progressive parietal cell loss, massive fundic hyperplasia, hypochlorhydria and hypergastrinemia, starting a few weeks after birth. These findings suggest that Slc26a9 expression is essential for parietal cell function and survival [4]. Parietal cell loss is a key step in the generation of a premalignant environment [8]. The importance of Slc26a9 expression in the gastric mucosa, as well as in parietal cells themselves, for the prevention of malignant transformation has not been previously studied. We therefore addressed the questions whether absence of Slc26a9 expression may promote gastric carcinogenesis in mice, whether a selective loss of Slc26a9 in parietal cells may be sufficient to induce metaplasia and, ultimately, GC. We also studied SLC26A9 expression during the development and progression of human GC and correlated it with prognosis. To explore its underlying mechanism, the effect of exogenous SLC26A9 expression on GC cell function was assessed.

## Materials and methods

For detailed additional method descriptions see Supplementary Materials.

### Animal models

The Slc26a9-deleted mouse strain used, whose establishment and characteristics have been described elsewhere [4, 5], is congenic on a S129/svj background. *Slc26a9*^*fl/fl*^ mice with a C57BL/6 J background were obtained from Cyagen Biosciences, China. *Atp4b-Cre* mice [9] were donated by Prof. Xiao Yang from the State Key Laboratory of Proteomics, China. *Slc26a9*^*fl/fl*^ mice were crossed with *Atp4b-Cre* mice to produce parietal cell-specific Slc26a9 knockout *Slc26a9*^*fl/fl*^*/Atp4b-Cre* mice with a mixed genomic background. The mice were bred and genotyped in accordance with the Institutional Animal Care and Use Committee (IACUC) at Hannover Medical School, Germany, and at Zunyi Medical University, China, respectively. All mice ranging from 8 days to 18 months old were age- and sex-matched. All experiments involving animals were approved by the Hannover Medical School and Zunyi Medical University committees on investigations involving animals and an independent committee assembled by the local authorities.

### Tissue microarray and primary human samples

A tissue microarray containing 90 cases of multiple human GC tissues (HStm-Ade180Sur-06) was obtained from Shanghai Outdo Biotech. Human samples, including normal gastric epithelium (*H. pylori* negativity was confirmed by histologic examination for *H. pylori* and a C14 urea breath test), and chronic atrophic gastritis (CAG) samples were obtained from the Endoscopy Center and 145 GC tissues from the Department of Pathology of the Affiliated Hospital of Zunyi Medical University, from January 2010 to January 2019. The study was performed in accordance with the Second Helsinki Declaration and was approved by the Human Subject Committee of the Affiliated Hospital of Zunyi Medical University, Zunyi, China. All patients involved provided written informed consent.

### Statistics

Statistic significances were analyzed by two-tailed Student’s t test or one-way ANOVA using SPSS 19.0 software (SPSS Inc., Chicago, IL, USA). Survival curves were plotted using the Kaplan–Meier method and compared using log-rank tests. All analyses were performed at least in triplicate. The results are reported as the means ± SD. Significance was defined as *p < 0.05, *p < 0.01, ***p < 0.001 or ****p < 0.0001; NS, not significant.

## Results

### Genetic deletion of Slc26a9 leads to spontaneous premalignant and malignant lesions in murine gastric mucosal epithelia

**Fig. 1:**
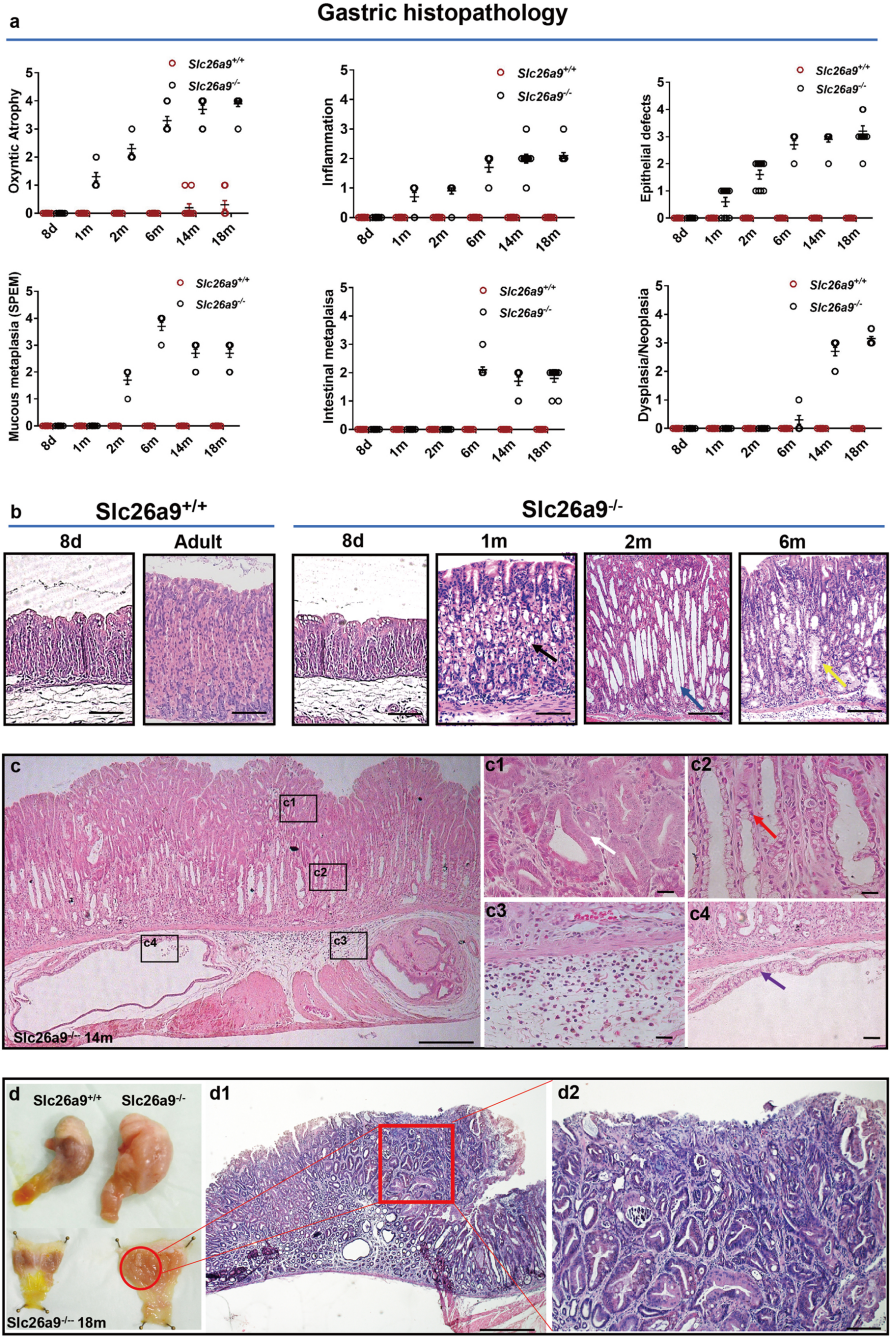
Slc26a9 deletion results in spontaneous gastric premalignant and malignant lesions. (**a**) Gastric histopathological scoring evaluation of *slc26a9*^+*/*+^ and *slc26a9*^*−/−*^ mice from 8 days to 18 months after birth based on Roger’s criteria. n = 10 in each series. (**b**) Histological comparisons of stomachs between *slc26a9*^+*/*+^ and *slc26a9*^*−/−*^ mice from 8 days to 6 months. The black arrow indicates parietal cell loss, the blue arrow indicates a dilated gland and the yellow arrow indicates SPEM. (**c**) *slc26a9*^*−/−*^ mice display all premalignant and malignant lesions in gastric epithelia at 14 months after birth at higher magnification (c1-c4). c1, HGIN (white arrow); c2, atrophy with mucosa cell metaplasia (red arrow); c3, inflammation; c4, gastritis cystica profunda (purple arrow). (**d**) Morphologic image of the stomach and its histology in *slc26a9*^*−/−*^ mice at 18 months. The stomach of *slc26a9*^*−/−*^ mice was approximately twice as thick as that of WM. d1, red box indicates moderately differentiated gastric carcinoma in gastric corpus epithelia; d2, higher magnification images for d1. Scale bars represent 400 μm (c and d1), 100 μm (b) and 50 μm (c1-c4 and d2)

The histopathology of gastric mucosae was investigated in *slc26a9*^*−/−*^ and wild-type littermates aged 8 days to 18 months using a murine gastric histopathology scoring system [10, 11] (Fig. 1a). We found that the gastric mucosae of *slc26a9*^−/−^ mice were not significantly different from those of wild-type mice (WM) at 8 days after birth. Parietal cell loss was observed at 1 month of age, oxyntic atrophy (parietal cell and chief cell loss) with elongated and dilated glands was observed at 2 months of age and mucous cell metaplasia, including spasmolytic polypeptide-expressing metaplasia (SPEM) and intestinal metaplasia (IM), was observed at 6 months of age (Fig. 1b). By 14 months of age, all of the Slc26a9-deficient mice (23/23) exhibited a severe gastric preneoplastic phenotype, including chronic atrophic gastritis (CAG), mucous cell metaplasia, profound cyst formation and high-grade intraepithelial neoplasia (HGIN), equivalent to early GC [10, 12] (Fig. 1c). Moderately differentiated GC (4/13) and HGIN (9/13) were found in the gastric corpus at 18 months of age (Fig. 1d). No submucosal invasion, lymph node or distant metastases or tumor formation in other organs were observed (data not shown).

### Identification of molecular markers for atrophy, SPEM, IM, epithelial defects, dysplasia and GC in Slc26a9-deficient gastric mucosa

**Fig. 2:**
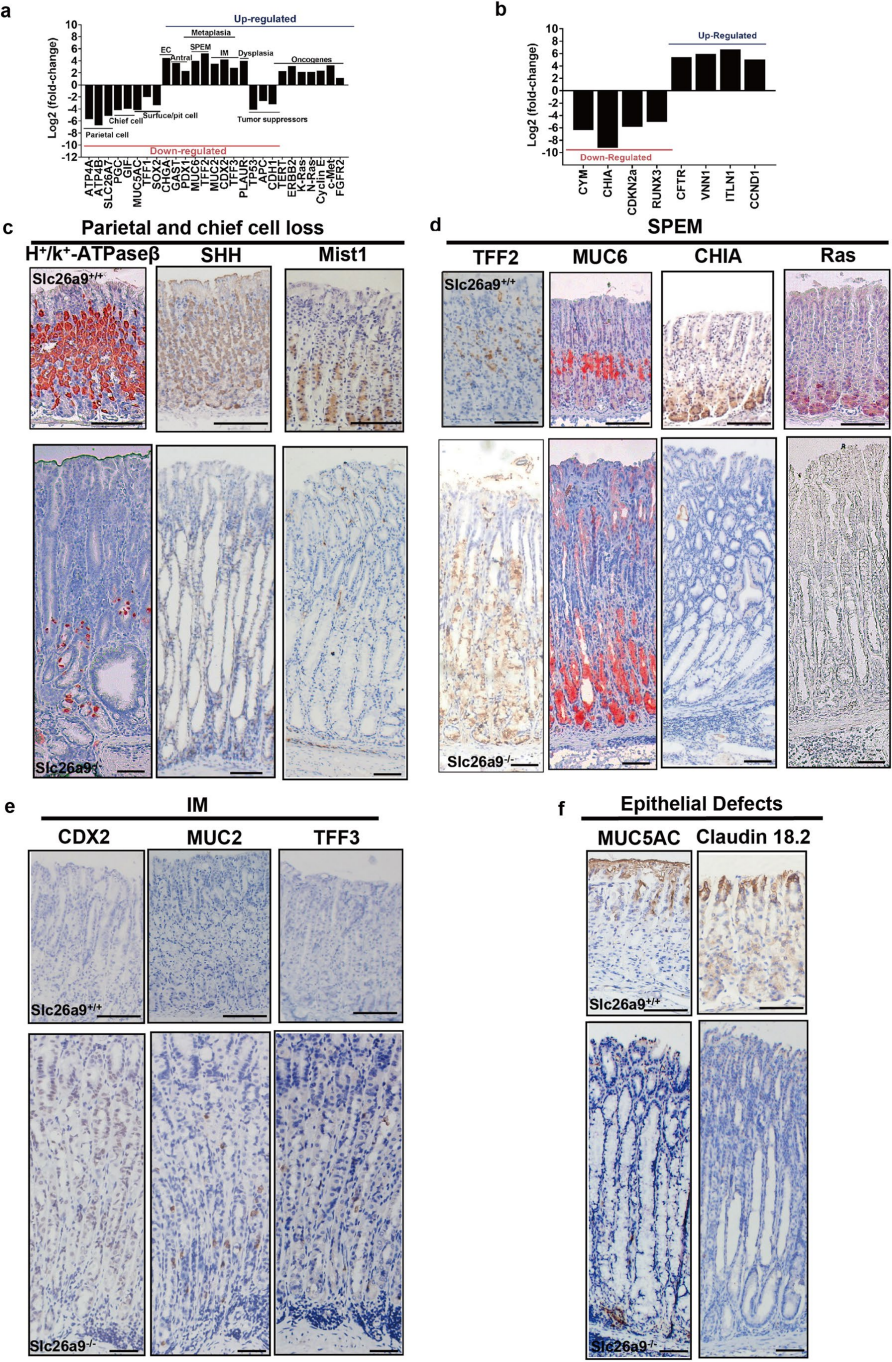
Identification of molecular markers for atrophy, SPEM, IM, epithelial defects, dysplasia and GC. (**a**) Log2 fold-changes of differentiation markers, including those of corpus and antral cell type-specific gene markers and metaplasia, dysplasia and GC markers. (**b**) Highly up- and downregulated genes in *slc26a9*^*−/−*^ mice at 14 months after birth. All data with black bars indicate significant gene changes with *p* values of 0.05 or less. (**c**-**f**) Representative IHC images of the gastric corpus of *slc26a9*^+*/*+^ and *slc26a9*^*−/−*^ mice at 6 months, including H^+^/K^+^-ATPaseβ, SHH and Mist1 (**c**), TFF2, MUC6 CHIA and Ras (**d**), CDX2, MUC2 and TFF3 (**e**) and MUC5AC and Claudin 18.2 (**f**). Scale bars represent 200 μm in *slc26a9*^+*/*+^mice and 100 μm in *slc26a9*^*−/−*^ mice

Using transcriptome analysis of *slc26a9*^*−/−*^ gastric mucosa, we found that the expression levels of differentiated gastric epithelial cell markers were significantly reduced, while several markers associated with both mucous cell metaplasia, including SPEM and IM, mucosal epithelial defect and dysplasia [13], were significantly upregulated. Based on microarray analyses, we found that known oncogenes in GC, including ErbB2, Cdkn2a, MAX, TERT, K-Ras, N-Ras, Cyclin E, c-Met and FGFR2, were upregulated at 14 months of age, whereas the tumor suppressors P53, CDH1 and APC were downregulated (Fig. 2a). The most highly up- and downregulated genes in *slc26a9*^*−/−*^ mice were Intelectin 1 (ITLN1) and CHIA, respectively (Fig. 2b). Previously, it has been found that the upregulation of ITLN1 is associated with GC onset in humans [14], and that downregulation of CHIA causes gastric atrophy [15]. We found that many of the top list genes were highly upregulated, such as CFTR, VNN1 and Cyclin D1 (CCND1), whereas CYM, CDKN2A and RunX3 were markedly downregulated (Fig. 2b). All of these genes have been previously implicated in gastric carcinogenesis [16–19].

Immunohistochemical analysis revealed similar changes. Compared with WT, *slc26a9*^*−/−*^ gastric mucosa exhibited significantly decreased expression levels of the gastric mucosal parietal cell markers H^+^/K^+^-ATPase β and Sonic hedgehog (Shh) and the chief cell marker Mist1 (Fig. 2c). These gastric mucosae also exhibited a disturbed surface epithelium, including downregulation of the MUC5AC and gastric Claudin 18.2 variants (Fig. 2f), a marked upregulation of specific SPEM markers, including TFF2 and MUC6, from the basement expanding to the upper gastric gland, and loss of acidic chitinase (CHIA) and Ras in the SPEM lesions (Fig. 2d). CDX2, MUC2 and TFF3 expression was found to be increased in *slc26a9*^*−/−*^ compared to WT gastric mucosa (Fig. 2e). Caudal-related homeobox transcription factor 2 (CDX2) is an intestine-specific transcription factor that has been implicated in the development of intestinal epithelial cells, where it regulates secretory cell lineage development, including MUC2 and TFF3 expression.

### Parietal cell-specific Slc26a9 deletion recapitulates the metaplastic alterations seen with complete Slc26a9 knockout

**Fig. 3:**
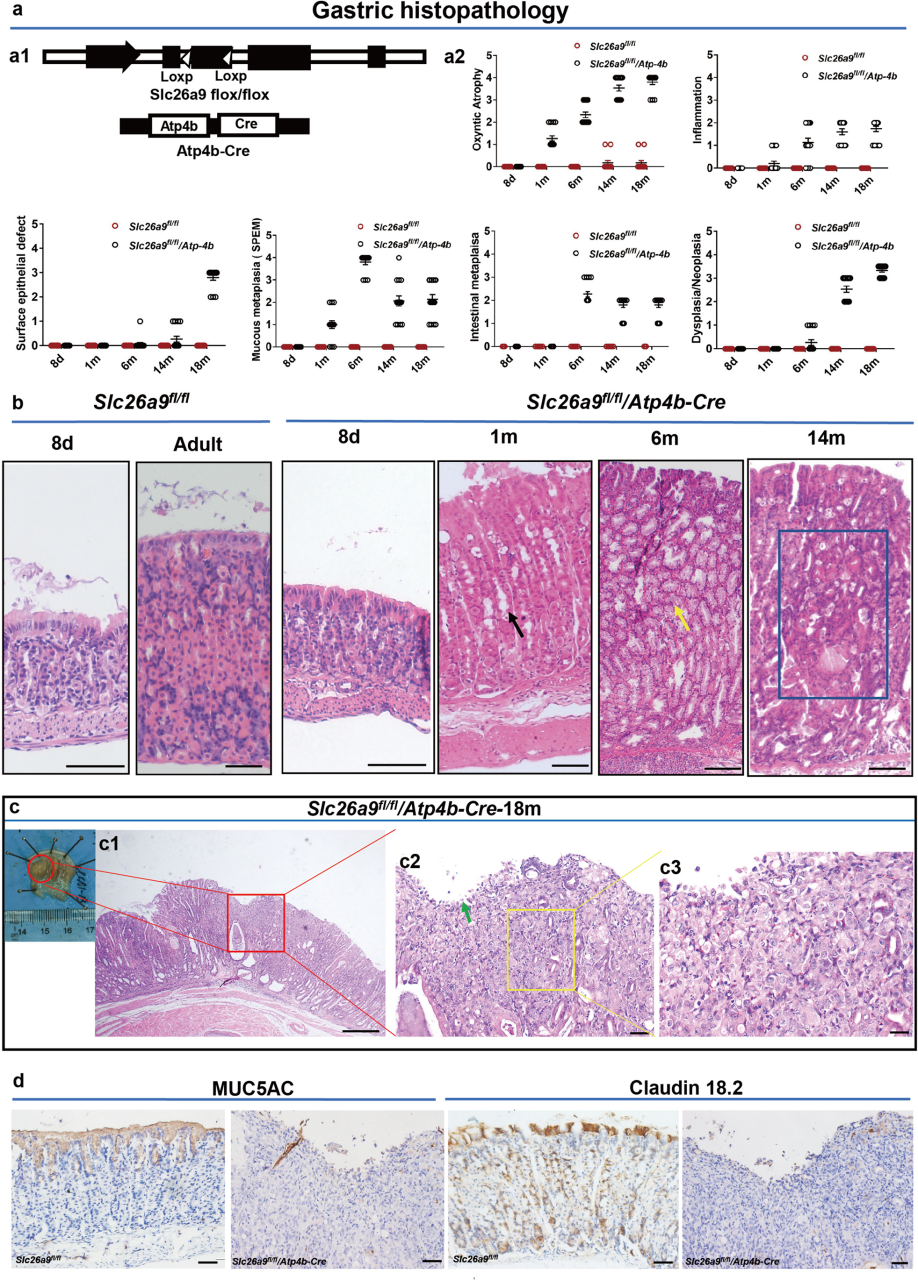
Specific deletion of Slc26a9 in parietal cells induces spontaneous precancerous and cancerous lesions in the murine stomach. (**a**) Gastric histopathological scoring evaluation of *Slc26a9*^*fl/fl*^ mice and *Slc26a9*^*fl/fl*^*/Atp4b-Cre* mice from 8 days to 18 months after birth (a1). Schematic diagram of the genome of Atp4b-Cre; Slc26a9 ^*fl/fl*^ mice (a2). Gastric histopathology scores for all mice at 8 days and 1, 6, 14 and 18 months are based on Roger’s criteria, n = 12–15 in each series. (**b**, **c**) Histological comparisons of *Slc26a9*^*fl/fl*^ mice and *Slc26a9*^*fl/fl*^*/Atp4b-Cre* mice from 8 days to 18 months after birth. (b) *Slc26a9*^*fl/fl*^*/Atp4b-Cre* mice display parietal cell loss (black arrow), SPEM (yellow arrow) and HGIN (blue box) at different ages. c1 is a whole image of a cancerous lesion. c2 is a higher magnification image of the red box of c1, showing an epithelial mucosa defect (green arrow) and poorly differentiated GC in gastric corpus epithelia (yellow box). c3 is a higher magnification image of c2. (**d**) Expression of the foveolar epithelial marker Muc5AC and the tight junction marker Claudin 18.2 (representative IHC staining images). Scale bars in b and d represent 200 μm in *Slc26a9*^*fl/fl*^ mice, 100 μm in *Slc26a9*^*fl/fl*^*/Atp4b-Cre* mice, 400 μm in c1, 50 μm in c2 and 20 μm in c3

Next, we explored whether the initial epithelial dysfunction is a loss of gastric surface barrier integrity, resulting in increased H^+^ back diffusion and secondary loss of parietal cells, or whether the primary event is a defect in parietal cell development. To this end, a parietal cell-specific Slc26a9-knockout mouse model was generated (Fig. 3a, Supplementary Fig. 1), after which gastric mucosal histopathology was investigated in *Slc26a9*^*fl/fl*^*/Atp4b-Cre* and *Slc26a9*^*fl/fl*^ littermates aged 8 days to 18 months. We found that the gastric mucosae of *Slc26a9*^*fl/fl*^*/Atp4b-Cre* mice were not significantly different from *Slc26a9*^*fl/fl*^ mice at 8 days after birth. A loss of parietal cells and oxyntic atrophy with mucous cell metaplasia was observed at 1 month and 6 months of age, respectively, whereas HGIN was observed at 14 months of age (Fig. 3b). Compared with *Slc26a9*^*fl/fl*^ mice, *Slc26a9*^*fl/fl*^*/ATP4b-Cre* mice exhibited significantly decreased expression levels of the parietal cell marker H^+^/K^+^-ATPase β in gastric mucosae (Supplementary Fig. 2a), but identical expression levels of the foveolar epithelial marker MUC5AC at different time points using IHC analysis (Supplementary Fig. 2b). Claudin 18.2 was expressed in the surface cells and parietal cells in *Slc26a9*^*fl/fl*^ mice, but was significantly decreased in the parietal cells and slightly decreased in the surface cells of *Slc26a9*^*fl/fl*^*/Atp4b-Cre* mice at 14 months of age. No changes were observed in gastric surface cells from 8 days to 6 months (Supplementary Fig. 2c). Nevertheless, poorly differentiated carcinoma was observed in *Slc26a9*^*fl/fl*^*/Atp4b-Cre* mice at 18 months (Fig. 3c), which was accompanied by loss of MUC5AC and Claudin 18.2 expression compared with that in *Slc26a9*^*fl/fl*^ littermates (Fig. 3d). These data indicate that parietal cell-specific Slc26a9 deletion is sufficient to initiate GC development.

### Parietal cell-selective Slc26a9 deletion results in dysregulated differentiation of stem and progenitor cells in an inflammatory environment

**Fig. 4:**
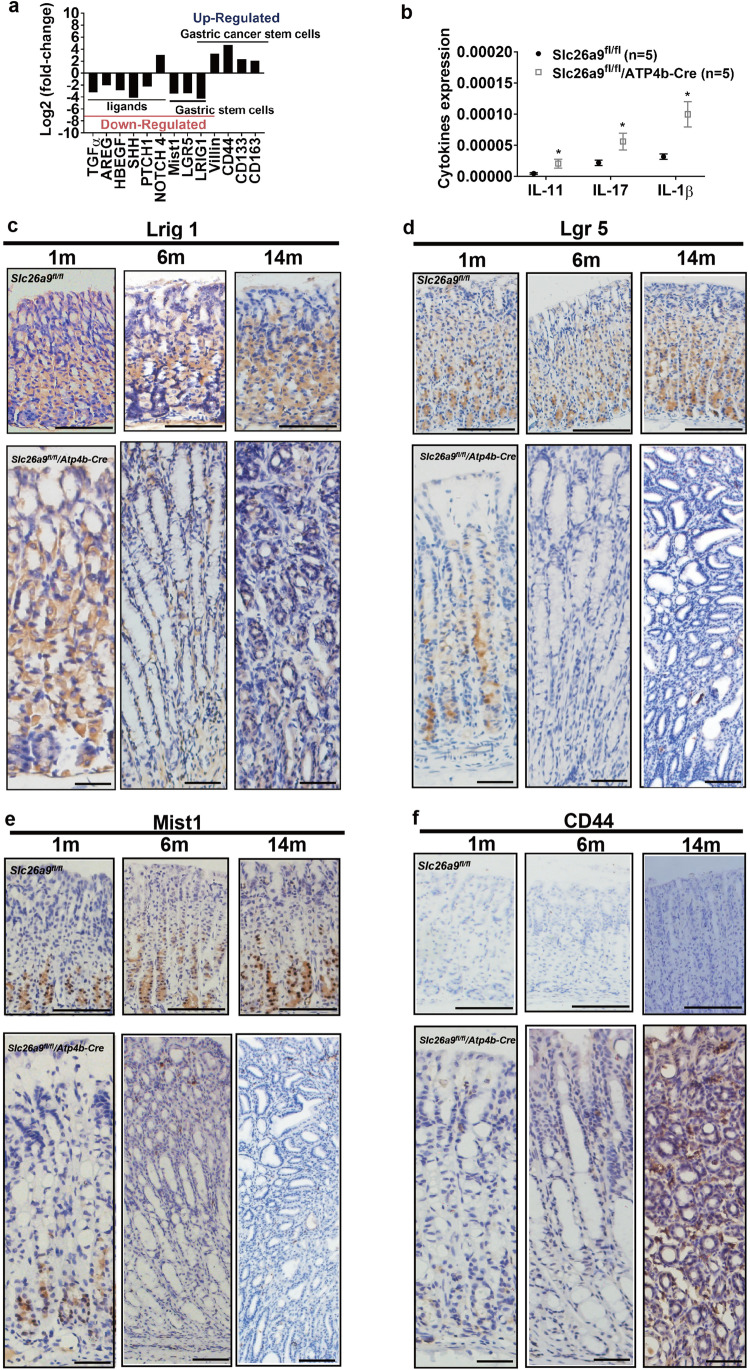
Slc26a9 deficiency causes dysregulated stem cell differentiation in an inflammatory environment. (**a**) Log2 fold-changes in multiple ligand genes, GSC and CSC markers in *Slc26a9*^*fl/fl*^*/Atp4b-Cre* mice at 14 months. All data with black bars indicate significant gene changes with *p* values of 0.05 or less. (**b**) mRNA expression of the proinflammatory cytokines IL-17, IL-11 and IL-1β in gastric tissues of *Slc26a9*^*fl/fl*^ mice and *Slc26a9*^*fl/fl*^*/Atp4b-Cre* mice. (**c**-**f**) Location and expression of multiple GSC markers, including Lrig1 (c), Lgr5 (d) and Mist1 (e)*,* as well as the CSC marker CD44 (f) in gastric mucosa of *Slc26a9*^*fl/fl*^ mice and *Slc26a9*^*fl/fl*^*/Atp4b-Cre* mice at different time points, determined by ISH and IHC (representative images). Scale bars represent 200 μm in *Slc26a9*^*fl/fl*^ mice and 100 μm in *Slc26a9*^*fl/fl*^*/Atp4b-Cre* mice

We found that loss of Slc26a9 expression resulted not only in a significant alteration of gastric epithelial cell differentiation (Fig. 2a), but also in the expression of multiple genes encoding ligands, including transforming growth factor-α (TGF-α), amphiregulin (AREG), heparin-binding EGF (HB-EGF), Shh, Ptch1 and Notch4 (Fig. 4a), which are secreted by parietal cells and function as critical regulators of cell differentiation in the gastric mucosa [20]. These alterations were accompanied by upregulation of the inflammatory cytokines IL-17, IL-11 and IL-1β, indicating spontaneous inflammation (Fig. 4b)*.* Gastric stem cell (GSC) markers, including Lrig1, Lgr5 and Mist1, were downregulated in *Slc26a9*^*fl/fl*^*/ATP4b-Cre* mice as determined by transcriptome analysis (Fig. 4a), which was confirmed by RNA in situ hybridization (ISH) and IHC at 1 month, 6 months and 14 months after birth (Fig. 4c-e). Previous studies showed that Lrig1 is expressed in both stem cells and parietal cells, implying possible roles for the Lrig1 protein in both cell types [21]. Lgr5, which functions as a stem cell marker of chief cell differentiation, was found to be restricted to a subpopulation of non-proliferative zymogenic chief cells at the base of the corpus gland [22, 23]. Mist1 is expressed in both chief cells and stem cells [24]. While the expression and localization patterns of Lrig1, Lgr5 and Mist1 in *Slc26a9*^*fl/fl*^ mice were similar to those reported in mouse gastric epithelium [21–24], the expression progressively decreased in *Slc26a9*^*fl/fl*^*/ATP4b-Cre* mice over the lifespan of the animals. Additionally, we observed enriched gene expression of surface markers, including CD44, CD133, CD163 and Villin, in *Slc26a9*^*fl/fl*^*/ATP4b-Cre* mice (Fig. 4a). The expression and progressive upregulation of CD44 were confirmed by IHC at 1 month, 6 months and 14 months after birth in *Slc26a9*^*fl/fl*^*/ATP4b-Cre* mice, whereas no CD44 expression was detectable in *Slc26a9*^*fl/fl*^ mice (Fig. 4f).

### Parietal cell-specific Slc26a9 deletion results in activation of the Wnt signaling pathway and induces hyperproliferation and apoptosis inhibition

**Fig. 5:**
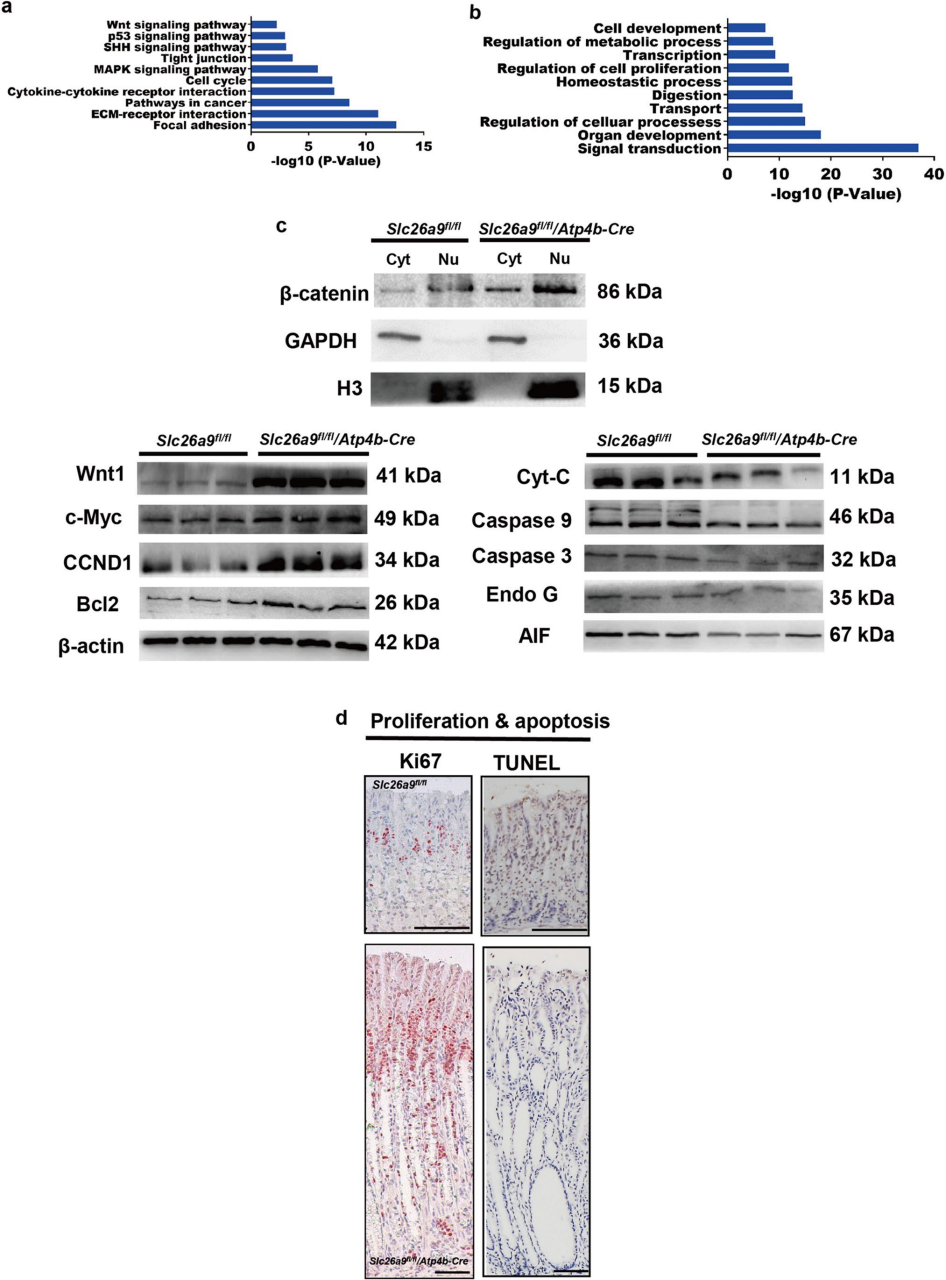
Slc26a9 deletion results in alteration of multiple signaling pathways and biological processes. (**a**) Top 10 signaling pathways and (**b**) top 10 biological processes deduced from overall gene expression levels. The data for a and b were obtained from 4 male *Slc26a9*^*fl/fl*^ mice and 4 male *Slc26a9*^*fl/fl*^*/Atp4b-Cre* mice. (**c**) Expression of Wnt signaling pathway target markers and apoptosis markers in *Slc26a9*^*fl/fl*^ mice and *Slc26a9*^*fl/fl*^*/Atp4b-Cre* mice. (**d**-**e**) Representative Ki67 and TUNEL IHC images. Scale bars represent 200 μm in *Slc26a9*^*fl/fl*^ mice and 100 μm in *Slc26a9*^*fl/fl*^*/Atp4b-Cre* mice

In the gastric mucosa of *Slc26a9*^*fl/fl*^*/ATP4b-Cre* mice at 14 months of age, upregulation of 494 genes and downregulation of 642 genes were found by RNA microarray analysis. The top 10 altered signaling pathways and biological processes with *p* values < 0.05 are shown in Fig. 5a and b. They were found to be associated with cell proliferation, differentiation, apoptosis, GSCs, transport activity, digestion, cell and organ development, and homeostasis as well as signal transduction, transcription and metabolism (Fig. 5a and b, Supplementary Tables 3 and 4), with pathways involving P53, tight junction, SHH, extracellular matrix (ECM) interaction, MAPK, cytokine–cytokine receptor interaction, cell cycle progression, Wnt, and focal adhesion as well as with alterations of numerous digestion- and metabolism-related gene families.

An imbalance in gastric epithelial cell proliferation and apoptosis has been reported to result in the development of GC, which is highly regulated by the Wnt signaling pathway [25]. Translocation of β-catenin to the nucleus is known to reflect activation of the Wnt pathway and to promote the transcription of downstream targets such as CCND1, c-Myc and Bcl2, which is associated with hyperproliferation and apoptosis suppression [25]. We found that the expression of Wnt1, β-catenin and downstream target genes, including CCND1, c-Myc and Bcl2, was significantly elevated in the *Slc26a9*^*fl/fl*^*/ATP4b-Cre* mice relative to controls, whereas cytochrome C (Cyt-C), cleaved Caspase 9, cleaved Caspase 3, apoptosis-inducing factor (AIF) and endonuclease G (Endo G) were downregulated, indicative of the suppression of caspase-dependent and caspase-independent apoptosis (Fig. 5c). In control mice, Ki67 and terminal deoxynucleotidyl transferase dUTP nick end labeling (TUNEL) assays showed that proliferating cells were localized to the isthmus region, and that apoptotic cells were present in the whole gland. In *Slc26a9*^*fl/fl*^*/ATP4b-Cre* mice, we found that proliferative cell areas were expanded to the entire length of the gastric glands, while apoptotic cells were significantly less numerous and confined to surface cells (Fig. 5d).

### SLC26A9 expression is progressively downregulated from CAG to GC in human gastric mucosa and is associated with a poor prognosis

**Fig. 6:**
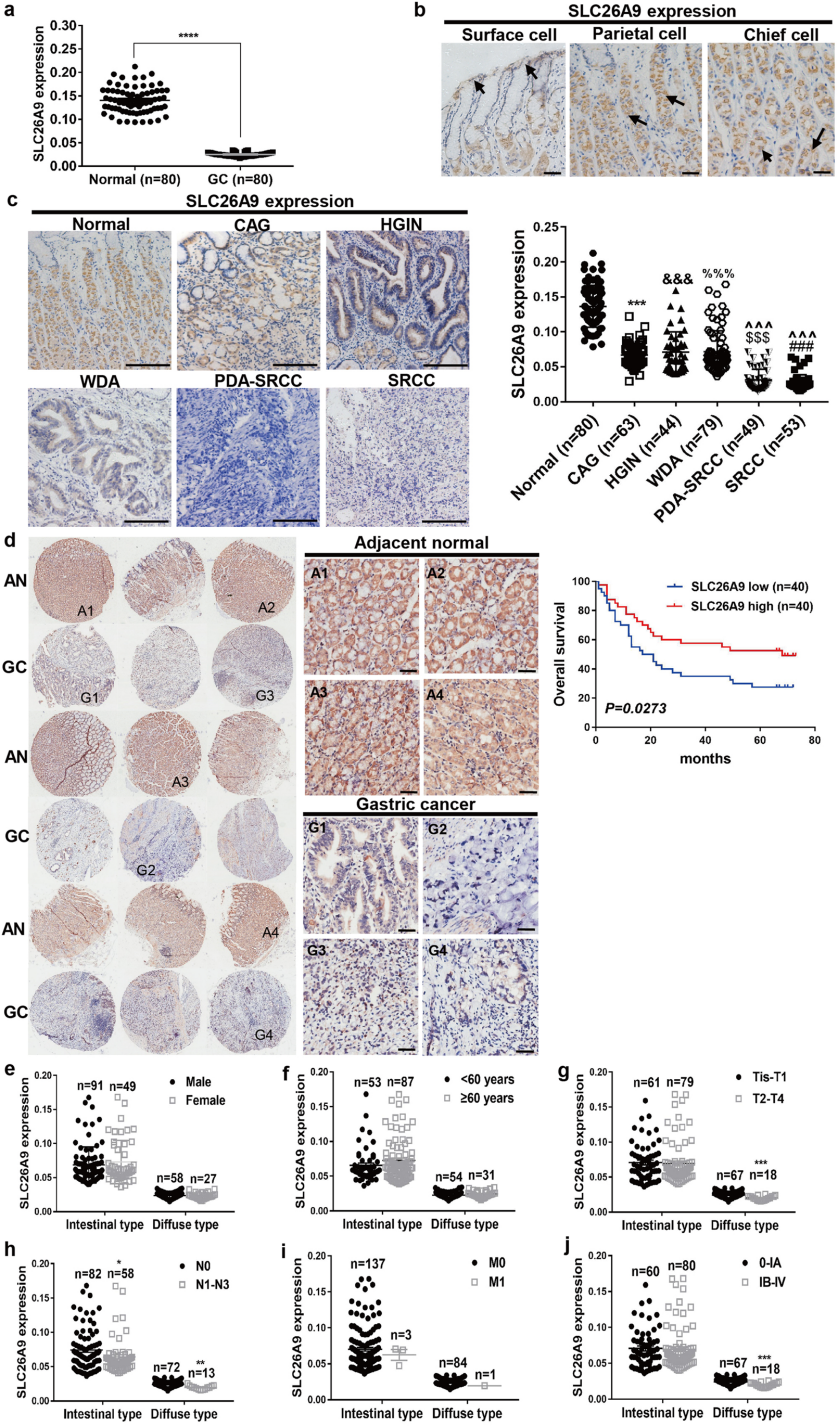
Downregulation of SLC26A9 is associated with the development and progression of human GC as well as a poor prognosis. (**a**) SLC26A9 mRNA expression in normal and GC tissues of the stomach detected by qRT-PCR. ****p < 0.0001, compared with normal. (**b**) Strong SLC26A9 expression in surface, parietal and chief cells of the normal human stomach detected by IHC analysis. (**c**) SLC26A9 expression is progressively downregulated from normal to CAG and HGIN to all types of GC as determined by IHC analysis. The left panel shows representative images, and the right panel shows a comparison of SLC26A9 expression levels between groups. ^***, &&&, %%%, $$$, ###^*p* < 0.001 compared with normal epithelia; ^^^*p* < 0.001 compared with HGIN and WDA groups. (**d**) Downregulation of SLC26A9 in GC determined by IHC in a tissue microarray is related to a poor prognosis. The left panel shows representative images of SLC26A9 expression in GC and adjacent normal tissues. The right panel shows the patient prognosis comparison between groups. (**e**-**j**) SLC26A9 expression in intestinal-type and diffuse-type GC tissues under different sex (e), age (f), tumor stage (g), lymph node (h), metastasis (i), and TNM stage (j) conditions. **p < 0.01, ***p < 0.001 and ****p < 0.0001 compared with relevant controls. Black arrows indicate different cellular locations of SLC26A9 in the normal stomach. Scale bars represent 100 μm (c) and 50 μm (b and d). WDA, well-differentiated adenocarcinoma; PDA-SRCC, poorly differentiated adenocarcinoma with signet ring carcinoma; SRCC, signet ring carcinoma

We next assessed SLC26A9 expression in human GC. Using qRT-PCR, we found that its expression in human GC tissues was significantly lower than that in normal gastric tissues (Fig. 6a). Subsequent IHC analysis revealed that SLC26A9 was predominantly localized in the cytoplasm and membrane of surface, parietal and chief cells in normal gastric epithelium (Fig. 6b) and progressively decreased from CAG to HGIN and to all types of GC (Fig. 6c). This finding indicates that progressive loss of SlC26A9 occurs in the human gastric mucosa across the different steps of epithelial dedifferentiation (gastritis, metaplasia, dysplasia and GC).

Next, we investigated the relationship between SLC26A9 expression in GC and patient survival by IHC analysis of a tissue microarray containing 90 GC tissues and 90 adjacent matched normal tissues; 80 tissues from each group were successfully stained and evaluated. After quantification of SLC26A9 protein expression in the tumor and adjacent normal tissues, the patient population was sorted based on SLC26A9 expression above (SLC26A9 high) or below (SLC26A9 low) the median. By doing so, we found that the patients in the SLC26A9 low-expression group had a lower overall survival than those in the SLC26A9 high-expression group (Fig. 6d).

Finally, we assessed SLC26A9 expression in diffuse- and intestinal-type GC tissues of 225 GC patients, including 80 GC tissue microarray samples and 145 GC tissue samples from the Department of Pathology of the Affiliated Hospital of Zunyi Medical University. We found that SLC26A9 expression was significantly higher in intestinal-type GCs than in diffuse-type GCs (Fig. 6c). In addition, we found that loss of SLC26A9 expression was associated with tumor stage, lymph node stage, metastasis stage and TNM stage of GC, but not with age or gender in the diffuse type group, which consisted of pure signet ring carcinomas and poorly differentiated adenocarcinomas with signet ring carcinoma. These differences were not observed in intestinal-type GC, which included HGIN, well-differentiated adenocarcinoma and moderately differentiated adenocarcinoma (Fig. 6e-j).

### Exogenous SLC26A9 expression in AGS cells increases AE2 expression, reduces WNT signaling, proliferation and migration, and enhances apoptosis

**Fig. 7:**
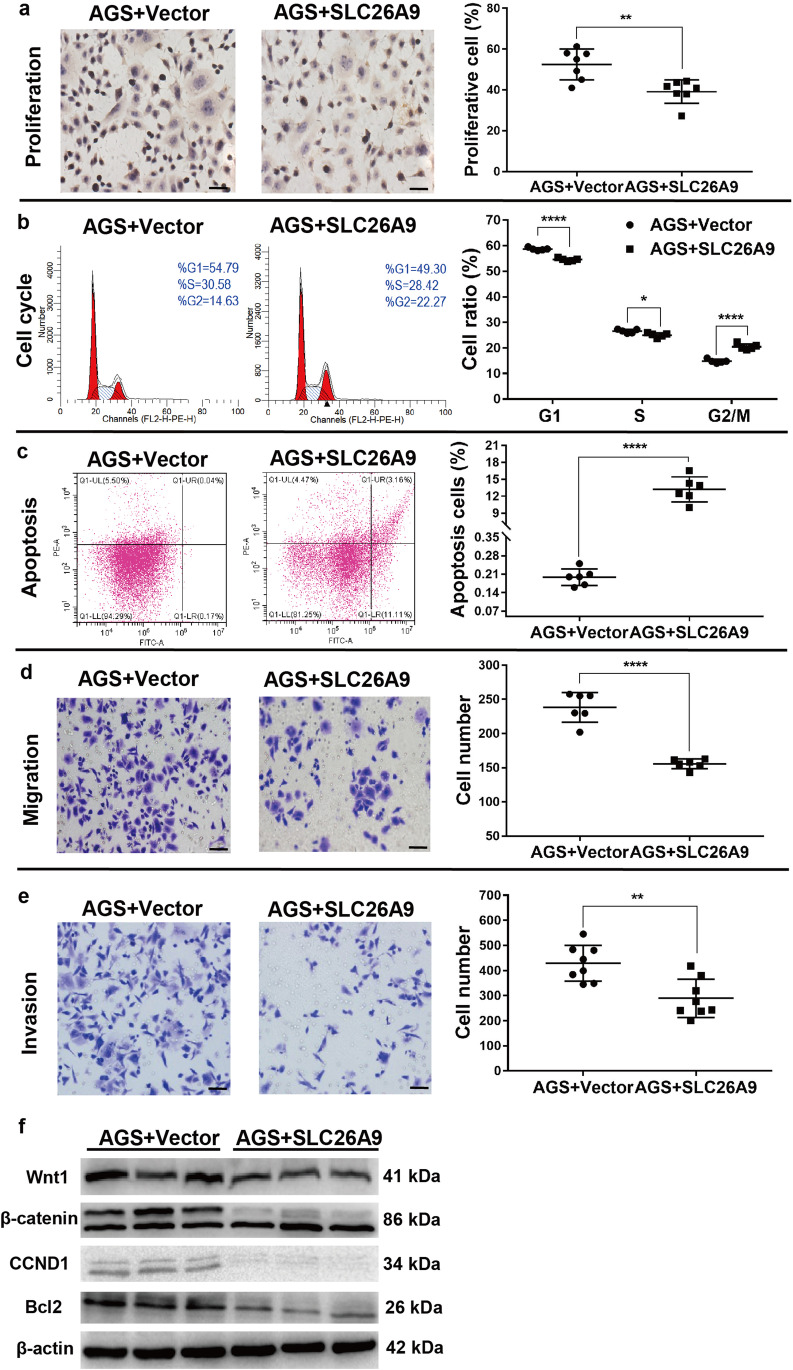
Effect of SLC26A9 overexpression in AGS cells on their biological behavior and the WNT signaling pathway. (**a**) Cell proliferation detected by Ki67 staining. (**b**) Cell cycle progression and (**c**) apoptosis determined by flow cytometry. (**d**) Cell migration and (**e**) invasion determined by Transwell assay. (**f**) Protein alterations in the WNT signaling pathway. Scale bars: 50 μm. **p < *0.01* compared with relevant controls, n = 6–8 in each series

To further explore the putative role of SLC26A9 in human GC, its expression and function were investigated in human GC cell lines. We found that SLC26A9 was abundantly expressed in the normal gastric epithelial cell line GES-1, but showed a low expression in the GC cell lines MKN45, AGS, SGC-7901, MKN28 and KATOIII, at both mRNA and protein levels. Among these GC cell lines, the lowest expression was observed in the poorly differentiated adenocarcinoma cell line AGS and the signet ring cell carcinoma cell line KATOIII, and the highest expression was observed in the well-differentiated adenocarcinoma cell line 7901 (Supplementary Fig. 3a). Next, we examined whether the exogenous expression of SLC26A9 in AGS cells resulted in altered proliferation and/or migration. We found that exogenous expression of SLC26A9 in AGS cells significantly reduced their proliferation compared with empty vector-transfected controls (Fig. 7a, Supplementary Fig. 3c and d), arrested the cell cycle at the G2/M checkpoint (Fig. 7b), and increased the percentages of early- and late-stage apoptotic cells, as determined by annexin V-FITC staining and flow cytometry (Fig. 7c). TUNEL staining confirmed late-stage apoptosis (Supplementary Fig. 3e). In addition, we found that exogenous SLC26A9 expressing AGS cells exhibited reduced migration and invasion capacities compared with control cells (Fig. 7d and e). Additionally, compared with the control, SLC26A9 overexpression in AGS cells inhibited Wnt1, β-catenin and CCDN1 expression, indicating that SLC26A9 overexpression may block hyperproliferation by inhibiting the Wnt signaling pathway (Fig. 7f).

To gain additional insight into the inverse correlation between SLC26A9 expression and cellular survival, clearly demonstrated in AGS cells, but also implicated by the findings in human and murine gastric cells, we measured the expression of the basolateral Cl^−^/HCO_3_^−^ exchanger AE2 (SLC4A2) in the CG cell lines as well as in SLC26A9-transfected AGS cells (Supplementary Fig. 4a-c). We found that the expression of AE2 was low in the GC cell lines tested, was significantly higher in the normal gastric cell line than in the GC cell lines (Supplementary Fig. 4a), and was upregulated in AGS cells after Slc26a9 transfection (Supplementary Fig. 4b and c). These data suggest that Slc26a9, via its function as a Cl^−^ exporter, may indirectly enhance HCO_3_^−^ export via AE2-mediated Cl^−^ recycling in exchange for HCO_3_^−^.

## Discussion

Our data indicate that genetic Slc26a9 deletion results in parietal cell loss, metaplastic transformation, and the development of premalignant and malignant lesions in the gastric epithelium in a mouse background susceptible to neoplastic transformation. The molecular and morphological features of this metaplastic/neoplastic transformation developed over the lifespan of the mice.

GC develops through a series of histological changes known as Correa’s cascade of gastric carcinogenesis. This classic sequence outlines a process of gastric carcinogenesis that represents a gradual transition from gastritis to mucosal atrophy, intestinal metaplasia, mucosal epithelial defects, dysplasia, intraepithelial neoplasia and invasive carcinoma [1, 2]. We found that *slc26a9*^−/−^ mice displayed histopathologic and molecular features from atrophy, metaplasia, mucosal barrier defects, and dysplasia to intraepithelial neoplasia over the lifespan of the mice.

In human gastric mucosal specimens of various pathologies, we found that SLC26A9 expression decreased progressively from chronic atrophic gastritis to GC. Within the GC specimens, SLC26A9 expression levels correlated with differentiation state and clinical outcome. We also studied SLC26A9 expression in a variety of GC cell lines and correlated its expression with cellular differentiation and behavior (Supplementary Fig. 3). Similar to the clinical specimens, we found that nonmalignant and well-differentiated carcinoma cells exhibited a higher SLC26A9 expression than nondifferentiated carcinoma cells.

These observations raised the question whether SLC26a9 expression loss is simply a sign of a loss of the polarized epithelial state of the cells or whether SLC26A9 functions as a “tumor suppressor” affecting cellular growth. A recent report showed that mammalian Slc26a9 is a Cl^−^ uniporter [26] that mediates Cl^−^ efflux after exogenous expression [27, 28]. We found that exogenous SLC26A9 expression in nondifferentiated, nonpolarized AGS cells reduced their proliferation and promoted their apoptosis. It is, therefore, feasible that a reduction in intracellular Cl^−^ concentration due to the presence of SLC26A9 in the plasma membrane controls growth. This notion was underscored by the analysis of AE2 expression in GC cells and SLC26a9-transfected AGS cells. Intracellular Cl^−^ ions have recently been recognized as important signaling molecules [29–31], regulating a wide variety of cellular processes, including proliferation, with high intracellular Cl^−^ levels promoting growth and low levels reducing growth [29]. It has long been known that loss of another epithelial Cl^−^ efflux protein, the Cl^−^ channel CFTR, which is highly expressed in the more distal parts of the GI tract, is associated with a strong increase in the incidence of colon cancer [32–34]. Recent work has shown that CFTR is highly expressed in intestinal stem cells (ISCs) and that CFTR deletion results in increased Wnt signaling in these cells due to pH-dependent stabilization of plasma membrane association of Wnt [35]. Although Slc26a9 has been described as a low HCO_3_^−^-conductive Cl^−^ transporter [26, 27], it has been shown that deletion of an apical Cl^−^ channel results in reduced activity of the cellular acid loader, which exports less HCO_3_^−^ because of the high intracellular Cl^−^ concentration [36]. As yet, nothing is known regarding the role of Slc26a9 in cellular anion regulation, but studies addressing this role appear feasible in the near future.

Parietal cell degeneration is a fairly uniform phenomenon in gastric meta/dysplasia models, in which the deleted gene or the inhibiting event is not necessarily parietal cell enriched. The inhibiting event can be toxic [37], infectious [11, 13], mutation of a tumor-suppressor gene in gastric progenitor cells [38], or deletion of a gene that is important in gastric barrier function, such as Claudin 18 [39], or for mucosal protection, such as TFF1 [40] or Muc5 [41]. Early work has suggested that loss of parietal cells by a toxin serves as a key event in the transformation of chief cells into metaplastic cells, and that SPEM metaplasia is a form of premalignancy, similar to IM [37]. However, the loss of parietal cells was later reported to be as insufficient for the development of gastric metaplastic lesions [42]. It was, therefore, of interest to find that parietal cell-specific deletion of Slc26a9 also resulted in gastric neoplasia. Based on this observation, we next selectively deleted Slc26a9 in cells expressing the β-subunit of gastric H^+^/K^+^-ATPase, which is the parietal cell precursor [43, 44], and in parietal cells. We found that the parietal cell-selective deletion of Slc26a9 resulted in the development of premalignant and malignant phenotypes, as observed in the stomachs of full-knockout mice. Of note, compared with the early reduction in the expression of Claudin 18.2 or Muc5AC, we found that a mucosal barrier defect occurred in *slc26a9*^−/−^ mice, and that *Slc26a9*^*fl/fl*^*/ATP4b-Cre* mice displayed intact gastric foveolar epithelia and surface epithelia at a young age. MUC5AC and Claudin 18.2 expression was lost in late adulthood when cancerous lesions were detected. This finding indicates that loss of Slc26a9 expression in parietal cells is sufficient to initiate the sequence of events that leads to neoplastic transformation.

Other mouse models exist in which deletion of an ion transporter results in achlorhydria [3, 8, 45]. Interestingly, not all of these models exhibit quantitative parietal cell loss [46]. However, loss of parietal cells has been observed in certain mouse models with achlorhydria, such as the AE2^−/−^ [47], *nhe4*^−/−^ [48], *nhe2*^−/−^ [49] and *kcne2*^−/−^ [50] models, and they all result in metaplastic changes in the gastric mucosa. Thus, the generation of mature parietal cells may serve as a key step in the regulation of epithelial cell differentiation in oxyntic glands, and their loss may result in SPEM. However, whether oxyntic atrophy and SPEM metaplasia will lead to neoplastic transformation appears to depend on additional factors [51].

One interesting mouse model that shares many features with the *slc26a9*^−/−^ mouse model is the *kcne2*^−/−^ model of gastric neoplasia [50]. KCNE2 is the ancillary subunit of the KCNQ1 potassium channel, which targets the complex to the parietal cell secretory membrane and serves as the K^+^ recycling channel during acid secretion [52, 53]. *Kcne2*^−/−^ mice are achlorhydric from young age on, and their gastric mucosae display most of the histological and molecular features of slc26a9^−/−^ mouse stomachs up till neoplasia formation. KCNE2 is also downregulated in human gastric carcinoma, and its overexpression has been found to result in a reduced proliferation of GC cells [54]. One similarity between Kcne2/Kcnq1 and Slc26a9, which distinguishes the two from NHE2, NHE4 and the H^+^/K^+^ ATPase, is that their deletion abolishes a pathway for solute exit followed by cell volume reduction, while the other transporters are operative in the process of volume increase; their deletion results in a shrunken state. It is well known that proliferation requires volume expansion and that overexpression of NHE1 is conducive to volume expansion and in supporting malignant growth [55]. Another ancillary subunit of the Kcnq1 channel, namely, Kcne3, has been found to be upregulated in the kcne2^−/−^ stomach and to result in targeting of the Kcnq1/Kcne3 complex to the basolateral membrane. Kcne3 deletion in kcne2^−/−^ mice aggravated fundic hyperplasia [56]. This latter finding suggests that even in a completely achlorhydric state, the existence of a basolateral K^+^ channel reduces proliferation, possibly by promoting cellular volume reduction. Loss of Slc26a9 may similarly result in a severe gastric phenotype, since this channel represents the predominant pathway for cellular anion release, resulting in cellular volume reduction. Obviously, this hypothesis requires further testing.

Additional insight into the molecular mechanisms that may enhance malignant cell behavior in the absence of Slc26a9 was obtained by studying the expression of the Cl^−^/HCO_3_^−^ exchanger AE2, which exchanges intracellular HCO_3_^−^ against extracellular Cl^−^ under physiological conditions and, thus, functions as an acid loader [57]. We found that overexpression of Slc26a9 in AGS cells resulted in a strong upregulation of endogenous AE2 expression. Likewise, we found that AE2 expression was strongly decreased in GC cells compared to nonmalignant gastric cells, in accordance with previous observations [58]. This decrease in AE2 expression could be secondary to lack of an apical Cl^−^ exit pathway (such as Slc26a9) and a resultant cellular Cl^−^ overload. Since AE2 functions as a cellular acid loader, low AE2 activity will result in a high intracellular pH, as has been shown for intestinal crypt cells [36]. A high steady-state pH favors proliferation in malignant cells [59], as well as in intestinal nonmalignant cells [60]. A decrease in steady-state pH_i_ as a consequence of the restoration of a Cl^−^ efflux pathway by Slc26a9 overexpression in AGS cells and a concomitant upregulation of an endogenous HCO_3_^−^ export pathway may be another mechanism explaining the increase in apoptosis and reduction in malignant behavior of AGS cells. Conversely, downregulated Cl^−^ recycling and increased pH_i_ levels may explain the malignant behavior in cancer cells with downregulated Slc26a9 and AE2 expression.

Apart from a potential specific function of the deleted Slc26a9 gene for gastric epithelial function, the loss of parietal cells and the ensuing hypo/achlorhydria that are uniformly present in many mouse models of oxyntic atrophy/metaplasia and neoplasia, will initiate a cascade of events that are conducive to neoplasia development. We found that Slc26a9 deletion-mediated parietal cell loss altered, not only the expression of markers of gastric epithelial cell differentiation, but also that of multiple genes that encode ligands secreted by parietal cells and function as critical growth factors in the gastric mucosa, including SHH, Notch4, TGF-α, ARGE and HB-EGF [61]. Impaired acid secretion results in hypergastrinemia, which markedly stimulates gastric foveolar proliferation [62] and promotes bacterial overgrowth that triggers the upregulation of inflammatory factors and promotes neoplastic transformation [63]. It has been found that complete suppression of acid secretion in WT mice kept in a barrier facility in individually ventilated cages by a three-week treatment with esomeprazole-containing chow resulted in increased proliferation of gastric epithelial cells and a significant increase in mucosal TNF-α mRNA expression (Brigitte Riederer, unpublished data). Clearly, the genetic background, specific housing conditions and the observation time play a role in neoplastic transformation occurring in a given mouse model.

In summary, we found that Slc26a9 deletion leads to dysregulated differentiation of stem and progenitor cells in an inflammatory environment that results in gastric neoplasia in mice. Multiple signaling pathways that are related to the regulation of cell proliferation, apoptosis and differentiation, as well as barrier integrity [13, 25, 64, 65] were found to be dysregulated. Loss of Slc26a9 expression triggered a number of molecular events, including the nuclear translocation of β-catenin and activation of the Wnt pathway, which resulted in gastric epithelial cell proliferation and apoptosis imbalance, as well as tumorigenicity. Selective ablation of Slc26a9 in parietal cells also resulted in malignant transformation, although the pathological alterations in the surface region appeared later than in the complete Slc26a9 knockout mice. We also found that Slc26a9 expression is progressively downregulated in human gastric disease from chronic gastritis to metaplasia and from well-differentiated GC to cancer with a low differentiation grade. Exogenous Slc26a9 expression in a GC cell line reduced its proliferative and induced its apoptotic rates. These data suggest that Slc26a9 may play a role beyond that of apical Cl^−^ channel in parietal cells during acid secretion and may be involved in the regulation of cellular growth and survival. Additional research into the molecular regulation of Scl26a9 appears necessary to unravel its exact function in organs that express this molecule.

## Supplementary Information

Below is the link to the electronic supplementary material.

Supplementary file1 (JPG 703 KB)

Supplementary file2 (JPG 3271 KB)

Supplementary file3 (JPG 1020 KB)

Supplementary file4 (JPG 541 KB)

Supplementary file5 (PDF 228 KB)

Supplementary file6 (PDF 468 KB)

Supplementary file7 (PDF 1323 KB)

## Data Availability

The datasets generated during and/or analyzed during the current study are available from the corresponding author upon reasonable request.

## Abbreviations

AG: Atrophic gastritis
AIF: Apoptosis-inducing factor
AREG: Amphiregulin
CAG: Chronic atrophic gastritis
CCND1: Cyclin D1
CHIA: Acidic chitinase
Cyt-C: Cytochrome C
EGFR: Epidermal growth factor receptor
Endo G: Endonuclease G
GC: Gastric cancer
GCS: Gastric cancer stem cell
GSC: Gastric stem cell
HGIN: High-grade intraepithelial neoplasia
HB-EGF: Heparin-binding EGF
IM: Intestine metaplasia
ISH: In situ hybridization
ITLN1: Intelectin 1
SPEM: Spasmolytic polypeptide-expressing metaplasia
TGF-α: Transforming growth factor-α
TUNEL: Transferase dUTP nick end labeling
WM: Wild-type mice
