# Supplementary figures and images for: SLC26A9 deficiency causes gastric intraepithelial neoplasia in mice and aggressive gastric cancer in humans
Source: Cell Oncol (Dordr). 2022 Apr 14;45(3):381–98. doi: 10.1007/s13402-022-00672-x (PMC9187568; doi:10.1007/s13402-022-00672-x)

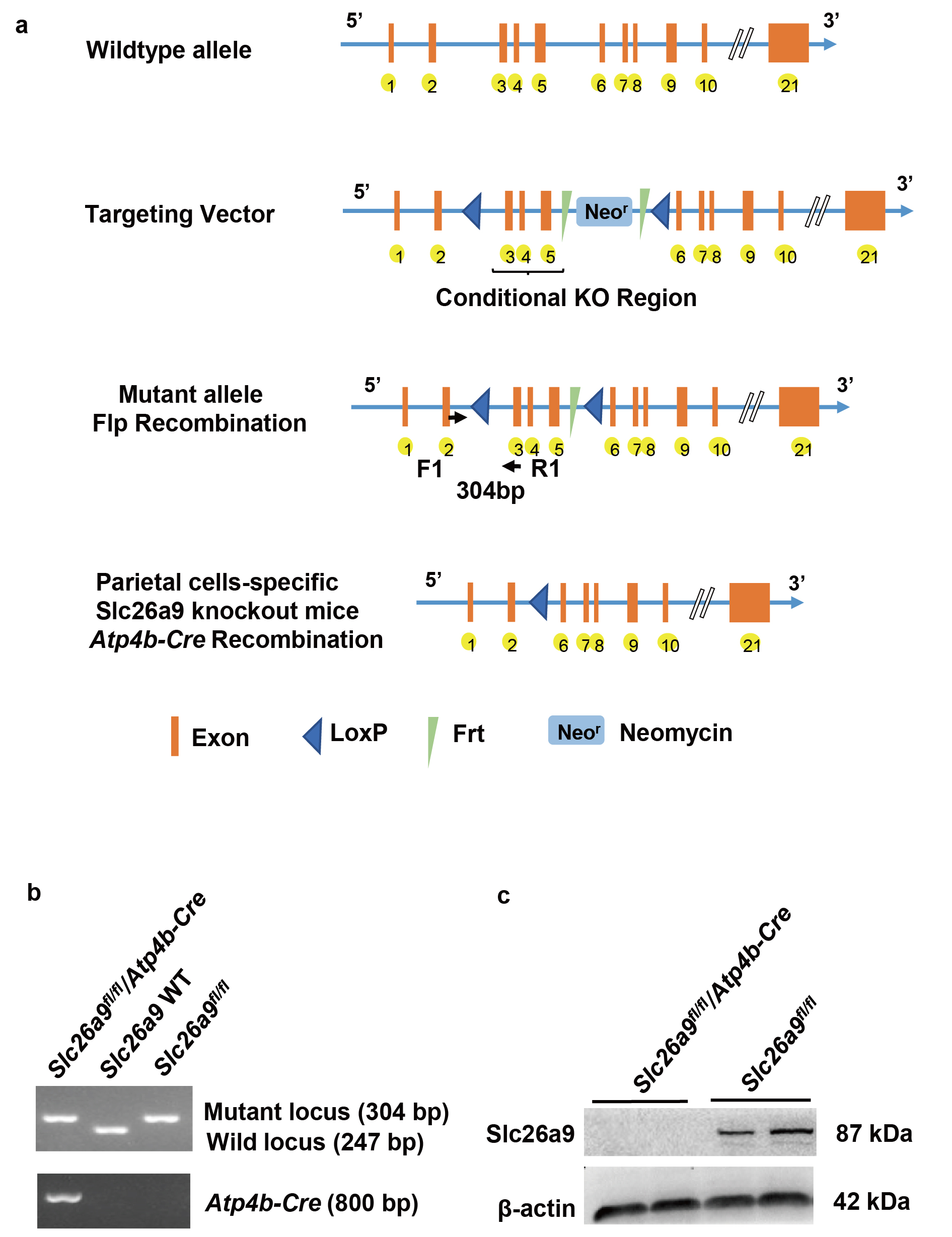

Supplement: Supplementary file 1 — Supplementary file1 (JPG 703 KB) [file 13402_2022_672_MOESM1_ESM.jpg]

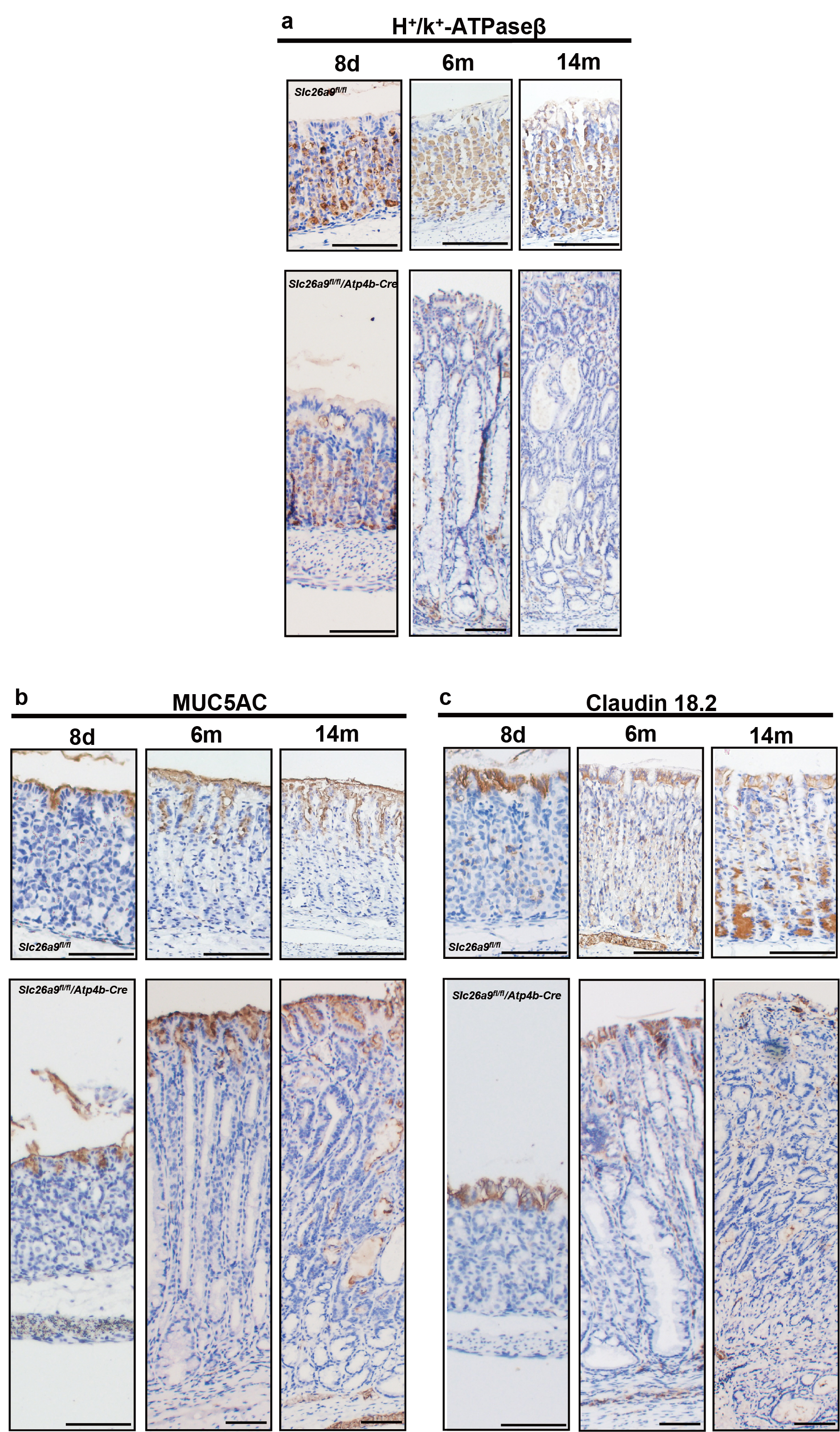

Supplement: Supplementary file 2 — Supplementary file2 (JPG 3271 KB) [file 13402_2022_672_MOESM2_ESM.jpg]

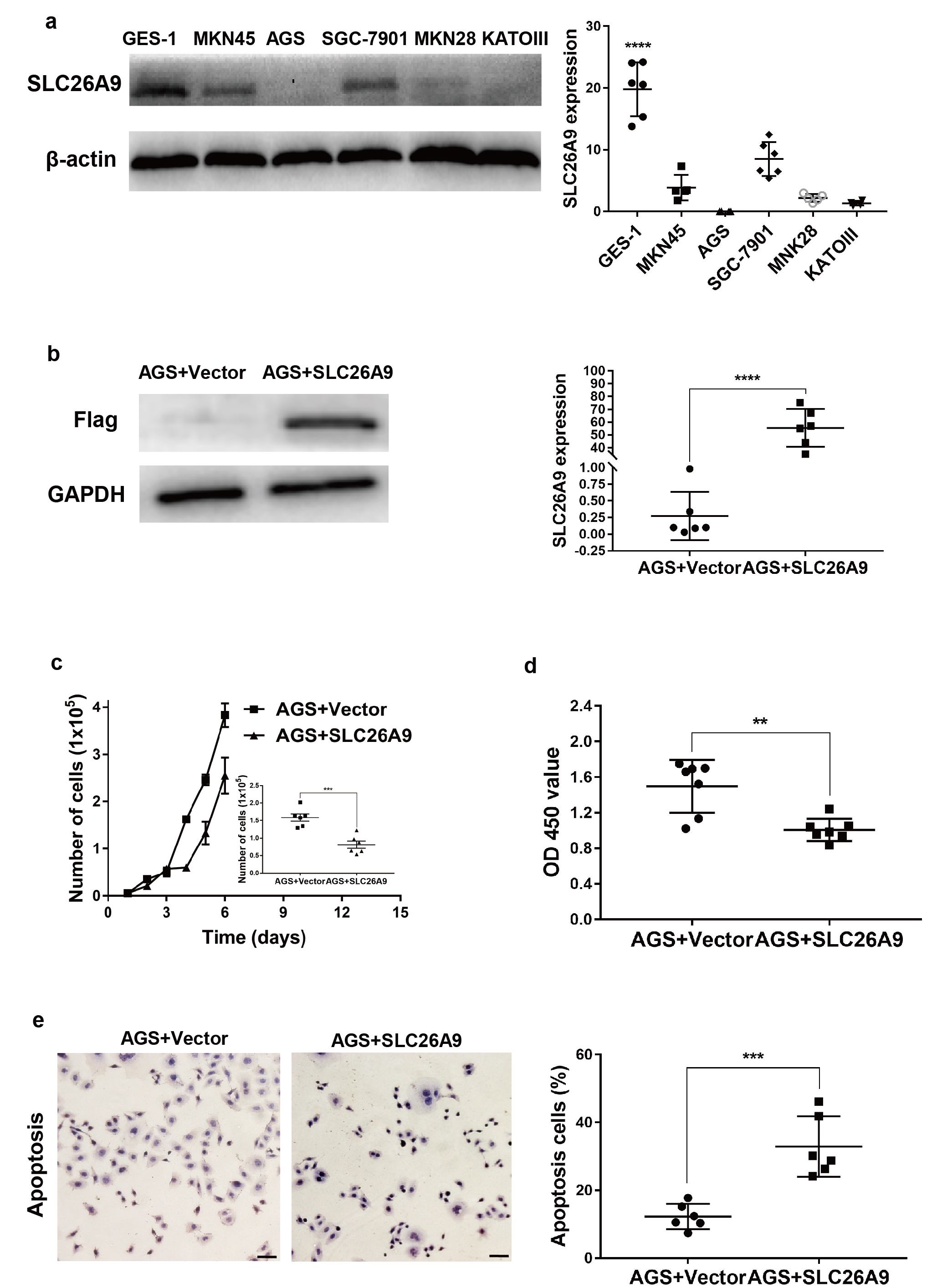

Supplement: Supplementary file 3 — Supplementary file3 (JPG 1020 KB) [file 13402_2022_672_MOESM3_ESM.jpg]

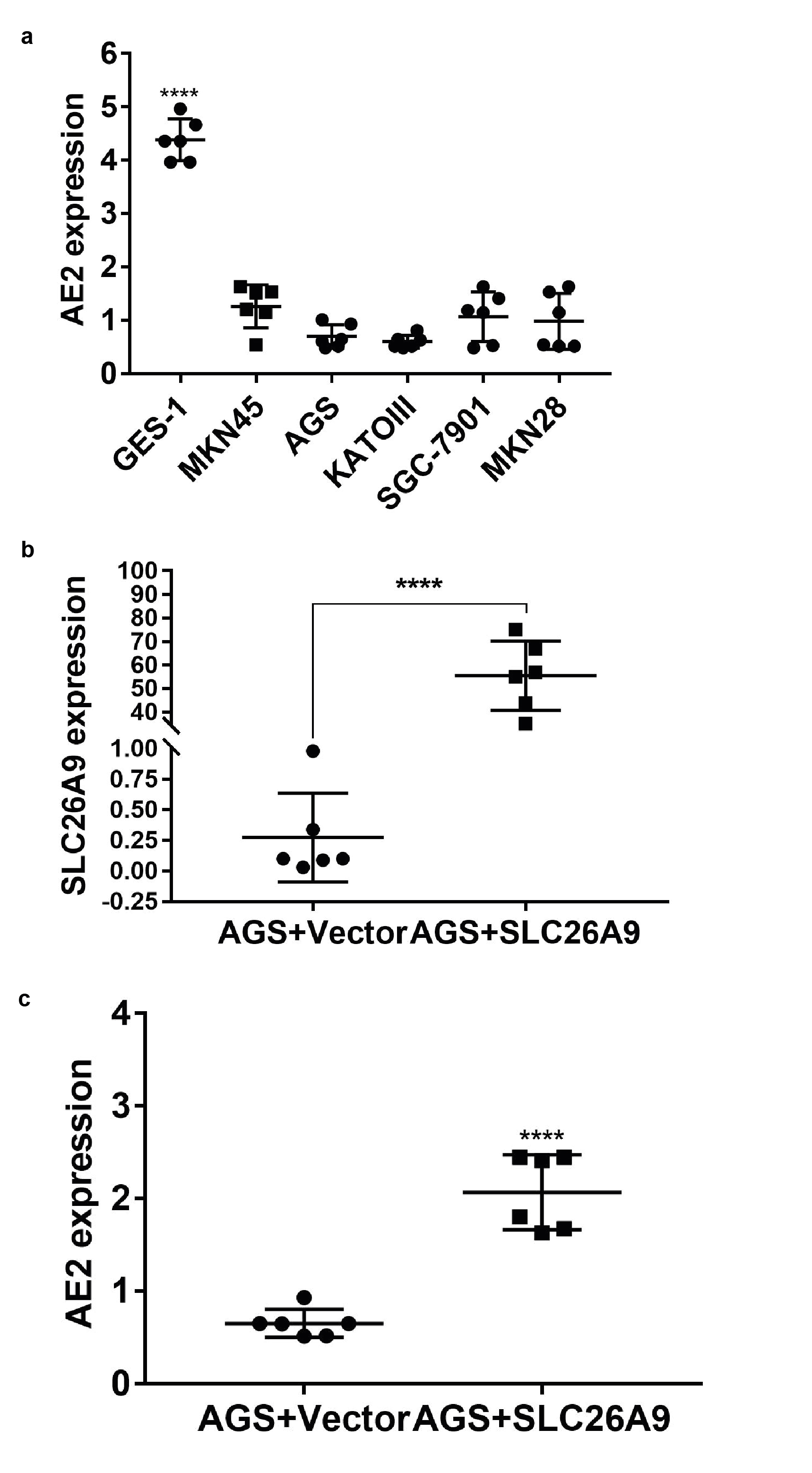

Supplement: Supplementary file 4 — Supplementary file4 (JPG 541 KB) [file 13402_2022_672_MOESM4_ESM.jpg]
