# Supplementary material for: SLC26A9 deficiency causes gastric intraepithelial neoplasia in mice and aggressive gastric cancer in humans
Source: Cell Oncol (Dordr). 2022 Apr 14;45(3):381–98. doi: 10.1007/s13402-022-00672-x (PMC9187568; doi:10.1007/s13402-022-00672-x)
Supplement: Supplementary file 5 — Supplementary file5 (PDF 228 KB) [file 13402_2022_672_MOESM5_ESM.pdf]

Supplementary Methods

Histological Study of the Stomach

Gastric tissue sections (2μm) were stained with hematoxylin and eosin as described previously by us [1]. The stomach was observed by using the murine gastric histopathology scoring system described by Rogers [2].

Specific Staining and Immunohistochemical (IHC) Analysis

Gastric tissue sections (2μm) of murine and human were prepared. Alcian Blue (AB, pH 2.5) and Periodic Acid-Schiff (PAS) staining was performed as previously described [3]. Lrig1 expression was detected by Lrig1 in situ hybridization kit as per the manufacturer's suggestions (MK2442, BOSTER Biological Technology, China). IHC was performed as described previously [1]. The different primary antibodies and dilutions are shown in Supplementary Table 1. Images were captured with an Olympus BX60 microscope (Olympus, Tokyo, Japan). Immunostained tissue was calculated by using Image-Pro Plus 6.0 (Media Cybernetics, Rockville, MD, USA), which has been widely used in biomedicine [4].

Gene Microarray Analysis

Total RNA of the stomach from Slc26a9 wildtype mice (WM) and knockout mice (KM) at 14 months of age were obtained for analysis with GeneChip® Mouse Genome 430 2.0 Array (Affymetrix, Santa Clara, CA, USA) as described previously [5], which covers transcripts and variants from 34,000 well characterized mouse genes. The chips were analyzed by using a GeneChip array scanner 3000 7 G (Affymetrix) and statistical analyses of changes in gene expression microarray probe by Program Packages of R Language. We chose those genes with a Log2 $\geq$ 2 (positive or negative), with an

adjusted *P*-value < 0.05 or less.

Western Analysis

Protein isolation, Western blot analysis and the quantification of target protein expression were performed as previously described [6], the list of primary antibodies is shown in Supplementary Table 1. Cytoplasmic and nuclear proteins were prepared with the ProteinExt Mammalian Nuclear and Cytoplasmic Protein Extraction Kit (TransGen Biotech) as previously described [7].

RNA Extraction and qRT-PCR

Total RNA isolation, qRT-PCR, and the quantification of target gene expression were performed as previously described [8]. The sequence of different genes is shown in Supplementary Table 2.

Cell Culture

The human gastric cancer cell lines (MKN28, KATOIII, SGC-7901, AGS and MKN45), and normal gastric mucosal cell line GSE1 were purchased form Chinese Academy of Sciences (Shanghai, China). The cells were cultured in DMEM with 10% FBS, and then placed in 37°C humidified incubators with 5% CO_2.

Lentiviral Transfection of AGS Cells

AGS cell were transfected with lentivirus that carries Slc26a9 gene fragment, or empty vector (Hanbio Biotechnology, China). A stable strain was selected by puromycin. qRT-PCR and western blot analyses were performed to validate the transfection efficiency.

Multiple Functional Analysis of GC Cell Ability

Cell proliferation (CCK-8 Cell Proliferation and Cytotoxicity Assay Kit, Solarbio, China), apoptosis (Annexin V-FITC Apoptosis Detection Kit & TUNEL Apoptosis Assay Kit, Solarbio, China) and cell cycle (DNA Content Quantitation Assay, Solarbio, China) were assessed according to the manufacturer's protocol. Migration and invasion assays were performed as described elsewhere [9].

Reference:

1. X. Liu, T. Li, B. Riederer, H. Lenzen, L. Ludolph, S. Yeruva, B. Tuo, M. Soleimani and U. Seidler, Loss of Slc26a9 anion transporter alters intestinal electrolyte and HCO3(-) transport and reduces survival in CFTR-deficient mice. Pflugers Arch **467**, 1261-1275 (2015) doi: 10.1007/s00424-014-1543-x
2. A.B. Rogers, Histologic scoring of gastritis and gastric cancer in mouse models. Methods Mol Biol **921**, 189-203 (2012) doi: 10.1007/978-1-62703-005-2_22
3. T. Li, X. Liu, B. Riederer, K. Nikolovska, A.K. Singh, K.A. Makela, A. Seidler, Y. Liu, G. Gros, H. Bartels, K.H. Herzig and U. Seidler, Genetic ablation of carbonic anhydrase IX disrupts gastric barrier function via claudin-18 downregulation and acid backflux. Acta Physiol (Oxf) **222**, e12923 (2018) doi: 10.1111/apha.12923
4. C.J. Wang, Z.G. Zhou, A. Holmqvist, H. Zhang, Y. Li, G. Adell and X.F. Sun, Survivin expression quantified by Image Pro-Plus compared with visual assessment. Appl Immunohistochem Mol Morphol **17**, 530-535 (2009) doi: 10.1097/PAI.0b013e3181a13bf2
5. H.S. Lee, E.Y. Kim and K.A. Lee, Changes in gene expression associated with oocyte meiosis after Obox4 RNAi. Clin Exp Reprod Med **38**, 68-74 (2011) doi: 10.5653/cerm.2011.38.2.68
6. B. Tuo, G. Wen, J. Wei, X. Liu, X. Wang, Y. Zhang, H. Wu, X. Dong, J.Y. Chow, V. Vallon and H. Dong, Estrogen regulation of duodenal bicarbonate secretion and sex-specific protection of human duodenum. Gastroenterology **141**, 854-863 (2011) doi: 10.1053/j.gastro.2011.05.044
7. X. Xu, A. Qimuge, H. Wang, C. Xing, Y. Gu, S. Liu, H. Xu, M. Hu and L. Song, IRE1α/XBP1s branch of UPR links HIF1α activation to mediate ANGII-dependent endothelial dysfunction under particulate matter (PM) 2.5 exposure. Sci Rep **7**, 13507 (2017) doi: 10.1038/s41598-017-13156-y
8. A. Pestana, R. Batista, R. Celestino, S. Canberk, M. Sobrinho-Simões and P. Soares, Comprehensive Assessment of TERT mRNA Expression across a Large Cohort of Benign and Malignant Thyroid Tumours. Cancers (Basel) **12**, (2020) doi: 10.3390/cancers12071846
9. E. Sjoberg, M. Meyrath, L. Milde, M. Herrera, J. Lovrot, D. Hagerstrand, O. Frings, M. Bartish, C. Rolny, E. Sonnhammer, A. Chevigne, M. Augsten and A. Ostman, A Novel ACKR2-Dependent Role of Fibroblast-Derived CXCL14 in Epithelial-to-Mesenchymal Transition and Metastasis of Breast Cancer. Clin Cancer Res **25**, 3702-3717 (2019) doi: 10.1158/1078-0432.CCR-18-1294
