# Supplementary material for: SLC26A9 deficiency causes gastric intraepithelial neoplasia in mice and aggressive gastric cancer in humans
Source: Cell Oncol (Dordr). 2022 Apr 14;45(3):381–98. doi: 10.1007/s13402-022-00672-x (PMC9187568; doi:10.1007/s13402-022-00672-x)
Supplement: Supplementary file 6 — Supplementary file6 (PDF 468 KB) [file 13402_2022_672_MOESM6_ESM.pdf]

Supplementary Fig. 1. *Slc26a9$^{fl/fl}$* mice were crossed with *Atp4b-Cre* mice to produce the parietal cells-specific Slc26a9 knockout in *Slc26a9$^{fl/fl}$/Atp4b-Cre* mice. (*a*) Generation of *Slc26a9$^{fl/fl}$/Atp4b-Cre* mice. Schematic representation of genomic structure of Slc26a9 wild type (WT) allele, Slc26a9 gene targeting construct, Slc26a9 targeted allele, and Slc26a9 null allele. Loxp, Frt and Neomycin sites are indicated. (*b*) Genotypes of *Slc26a9$^{fl/fl}$/Atp4b-Cre,* Slc26a9 WT and *Slc26a9$^{fl/fl}$* mice were determined by PCR amplification. Slc26a9 WT, *Slc26a9$^{fl/fl}$*, and *Atp4b-Cre* alleles were evidenced by amplification in 247bp, 304bp, and 800bp fragments, respectively. (*c*) Slc26a9 expression in the stomach of *Slc26a9$^{fl/fl}$/Atp4b-Cre* mice and *Slc26a9$^{fl/fl}$* mice were detected by Western blot. Loxp, Locus of X-overP1; Cre, Causes recombination; Frt, Flp recognition target; Neo^r, Neomycin.

Supplementary Fig. 2. IHC analysis of *Slc26a9$^{fl/fl}$* mice and *Slc26a9$^{fl/fl}$/Atp4b-Cre* mice at different time after birth. Expression of parietal cell marker H^+/K^+-ATPase β (*a*), foveolar epithelia marker Muc5AC (*b*) and tight junction marker Claudin 18.2 (*c*). Yellow arrows indicate the normal location and expression of different markers. Black arrows indicate the abnormality. Scale bars represent 200μm in all *Slc26a9$^{fl/fl}$* mice and all staining at 8 days, and represent 100μm in *Slc26a9$^{fl/fl}$/Atp4b-Cre.*

Supplementary Fig. 3. Expression and function of SLC26A9 in GC cells. *(a*) Protein and mRNA expression of SLC26A9 in the normal gastric epithelia cell line GES-1 and different type of GC cell lines. ***P*<0.01, compared with different type

of GC cell lines. (*b*) AGS cell were transfected with lentivirus that carries SLC26A9 gene fragment, or empty Vector. A stable strain was selected by puromycin and confirmed by western blot and qRT-PCR analyses to validate the repressive efficiency. GC cell growth and apoptosis were investigated by cell growth curve (*c*) and CCK8 (*d*) assay, as well as TUNEL staining (*e*), respectively, in AGS cells repressing SLC26A9 and empty vector control. ***P*<0.01 and *****P*<0.0001, compared with relevant controls. *Scale bars:* 50μm.

Supplementary Fig. 4. Relationship of SLC26A9 and AE2 mRNA expression in human GC cells. *(a)* AE2 in the normal gastric epithelia cell line GES-1 and different type of GC cell lines. *****P*<0.001, compared with different type of GC cell lines *(b)* Heterologous lentiviral overexpression of Slc26a9 results in strong upregulation of Slc26a9 mRNA expression and in *(c)* a strong increase in mRNA expression of endogenous AE2 in the Slc26a9-transfected cells. *****P*<0.001, compared with relevant control.

Supplementary Table 1. List of antibodies and staining conditions for tissue IHC

Antibody/Probe	Catalog #	Vendor	Antigen dilution
**Primary Antibodies to**:			
AIF	ab32516	Abcam	1:1000*
Bcl2	ab59348	Abcam	1:1000*
Caspase3	#9661	Cell Signaling Technology	1:1000*
Caspase9	ab202068	Abcam	1:2000*
CCND1	#55506	Cell Signaling Technology	1:1000*
CD44	ab157107	Abcam	1:1000
Claudin 18.2	ab222512	Abcam	1:500
c-Myc	bs-0842R	Bioss Antibodies	1:2000*
Cytochrome C	ab133504	Abcam	1:5000*
Endo G	ab9647	Abcam	1:1000*
E-cadherin	ab76055	Abcam	1:100; 1:500*
Flag-Tag	T9953	Affinity Research	1:25000*
GAPDH	#HC301-01	TransGen Biotech	1:5000*
H3	ab1791	Abcam	1:5000*
H^+/K^+-ATP-β	A10106	Abclonal	1:100
ki67	ab8191	Abcam	1:50
Lrg5	abs120810	Absin	1:400
Mist1	#14896	Cell Signaling Technology	1:100
MUC2	ab76744	Abcam	1:100
MUC5AC	ab3649	Abcam	1:100
MUC6	Orb253514	Biorbyt	1:100
N-cadherin	ab18203	Abcam	1:1000*
SHH	ab86462	Abcam	1:1000
Slc26a9	HPA-051485	Sigma-Aldrich	1:50
SLC26A9	NBP1-59514	Novus Biologicals	1:2000*
Snail	ab180714	Abcam	1:2000*; 1:200
TFF2	ab203237	Abcam	1:500
Vimentin	ab92547	Abcam	1:500; 1:2500*
Wnt1	ab15251	Abcam	1:500*
ZO-1	61-7300	Thermofisher Scietific	1:125; 1:500*
CHIA	ab72309	Abcam	1:1000
R-Ras	sc-523	Santa Cruz Biotechnology	1:200
CDX2	ab76541	Abcam	1:1000
TFF3	ab202967	Abcam	1:200
β-actin	#HC201	TransGen Biotech	1:5000*
β-catenin	ab32572	Abcam	1:10000*

List of antibodies was used in this study, * For Western Blot, others for IHC.

Supplementary Table 2. List of primers of multiple genes was used in this study

**RT-qPCR**		
**Gene (Mouse)**	**Forward sequence**	**Reverse sequence**
IL-17	GCGATCATCCCTCAAAGCTC	TCTTCATTGCGGTGGAGAGT
IL-6	ACTTCACAAGTCCGGAGAGG	TGCAAGTGCATCATCGTTGT
IL-1β	AGCTTCAAATCTCGCAGCAG	TCTCCACAGCCACAATGAGT
β-actin	CCTCTATGCCAACACAGTGC	GCTAGGAGCCAGAGCAGTAA
**Gene (Human)**	**Forward sequence**	**Reverse sequence**
AE2	ACCCTCATGTCAGACAAGCAA	TCTTGAGCATCTGGCGCT
SLC26A9	CCCAGGACACAACTTCCAA	AGGCACTCTGTAGGCAGCAT
β-actin	TGGCACCCAGCACAATGAA	CTAAGTCATAGTCCGCCTAGAAGCA
**PCR**		
**Parietal cells-specific knockout mice primers**	**Forward sequence**	**Reverse sequence**
Region 1	TGCAGATGCCGTTCTACCGAAT	TCTCTCAGGTGAGGGAAAGGCAAA
Cre	GCAGATAGCAAGCAAGCTCCAACC	GGATTAACATTCTCCCACCGTCAG

Supplementary Table 3. List of gene fold-change (log2) for different gene families, Data are for Slc26a9-deficient versus wild mice at 14 months after Birth

Gene family	Log2	Gene Name	Gene function
Up-regulation			
**Chitinase-like protein family**			
Chil3	3.350899	Chitinase-like protein 3	This gene family has low chemotactic activity for eosinophils. May play a role in inflammation and allergy.
Chil4	4.118467	Chitinase-like protein 4	
**Heat shock protein family**			
Hspa1a	4.354216	Heat shock protein family A (Hsp70) Member 1a	This gene family play important roles in carcinogenesis via the regulation of angiogenesis, cell proliferation, apoptosis, migration, invasion and metastasis.
Hspa1b	3.578588	Heat shock protein family A (Hsp70) Member 1b	
Hspa4	2.0558	Heat shock protein family A (Hsp70) Member 4	
Hspa4L	2.59846	Heat shock protein family A (Hsp70) Member 4 Like	
Hspa8	2.939886	Heat shock protein family A (Hsp70) Member 8	
**Prolactin family**			
Prl2c2	5.239832	Prolactin family 2, subfamily c, member 2	This gene family functions as a polypeptide hormone and potent multifunctional cytokine with a broad range of biological effects, including water and salt balance, lactogenesis, cell proliferation, survival, apoptosis and differentiation, T-cell immunity, pancreatic β-cell function, hematopoiesis, and adipogenesis
Prl2c3	5.239832	Prolactin family 2, subfamily c, member 3	
Prl2c4	5.239832	Prolactin family 2, subfamily c, member 4	
Down-regulation			
**Major urinary protein family**			
MUP1	-3.18704	Major urinary protein1	This gene family functions as binding pheromones that are released from drying urine of males, and thus stabilize them to allow slow release into the air from urine marks. May protect pheromones from oxidation. And binds the pheromone analog 2-sec-butyl-4,5-dihydrothiazole (SBT) *in*
MUP2	-3.18704	Major urinary protein2	
MUP3	-3.03246	Major urinary protein3	
MUP7	-3.18704	Major urinary protein7	
MUP8	-3.18704	Major urinary protein8	
MUP9	-3.18704	Major urinary protein9	
MUP10	-3.18704	Major urinary protein10	
MUP11	-3.03246	Major urinary protein11	

MUP12	-3.18704	Major urinary protein12	*vitro.*
MUP13	-3.18704	Major urinary protein13	
MUP14	-3.18704	Major urinary protein14	
MUP15	-3.18704	Major urinary protein15	
MUP16	-3.18704	Major urinary protein16	
MUP19	-3.18704	Major urinary protein19	
**Pancreatic triacylglycerol lipase family**			
PNLIP	-2.2616	Pancreatic Lipase	This gene family positive regulates triglyceride lipase activity and plays an important role in fat metabolism, intestinal cholesterol absorption, lipid digestion and metabolic process.
PNLIPRP1	-3.29538	Pancreatic Lipase related protein 1	
PNLIPRP2	-2.97507	Pancreatic Lipase related protein 2	
**Amylase Alpha 2A family**			
AMY2A1	-7.19922	Amylase Alpha 2A 1	This gene family catalyzing the first step in digestion of dietary starch and glycogen. Among its related pathways are galactose metabolism and digestion of dietary carbohydrate, dysfunction of this gene family is associated with pancreatitis onset.
AMY2A2	-7.19922	Amylase Alpha 2A 2	
AMY2A3	-7.19922	Amylase Alpha 2A 3	
AMY2A4	-7.19922	Amylase Alpha 2A 4	
AMY2A5	-7.19922	Amylase Alpha 2A 5	

Supplementary Table 4. List of gene fold-change (log2) for signaling pathways and biological processes for Figure 5A and 5B, Data are for Slc26a9-deficient versus wild mice at 14 months after Birth.

**Signaling pathways**		
Gene Set Name	Description	-Log10 (P-value)
KEGG_WNT_SIGNALING_PATHWAY	Wnt signaling pathway	2.239577517
KEGG_P53_SIGNALING_PATHWAY	p53 signaling pathway	2.928117993
BIOCARTA_PTC1_PATHWAY	Sonic Hedgehog (SHH) Receptor Ptc1 Regulates cell cycle	3.036212173
KEGG_TIGHT_JUNCTION	Tight junction	3.630784143
KEGG_MAPK_SIGNALING_PATHWAY	MAPK signaling pathway	5.790484985
KEGG_CELL_CYCLE	Cell cycle	7.059483515
KEGG_CYTOKINE_CYTOKINE _RECEPTOR_INTERAERACTION	Cytokine-cytokine receptor interaction	7.246416941
KEGG_PATHWAYS_IN_CANCER	Pathways in cancer	8.54668166
KEGG_ECM_RECEPTOR_INTERACTION	ECM-receptor interaction	11.04527521
KEGG_FOCAL_ADHESION	Focal adhesion	12.61978876
**Biological processes**		
Gene Set Name	Description	-Log10 (P-value)
CELL_DEVELOPMENT	Genes annotated by the GO term GO:0048468. The process whose specific outcome is the progression of the cell over time, from its formation to the mature structure. Cell development does not include the steps involved in committing a cell to a specific fate.	7.327902142
REGULATION_OF_METABOLIC_PROCESS	Genes annotated by the GO term GO:0019222. Any process that modulates the frequency, rate or extent of the chemical reactions and pathways within a cell or an organism.	8.815308569
TRANSCRIPTION	Genes annotated by the GO term GO:0006350. The synthesis of either RNA on a template of DNA or DNA on a template of RNA.	9.230622674
REGULATION_OF_CELL_PROLIFERATION	Genes annotated by the GO	11.84771166

	term GO:0042127. Any process that modulates the frequency, rate or extent of cell proliferation.	
HOMEOSTATIC_PROCESS	Genes annotated by the GO term GO:0042592. The biological processes involved in the maintenance of an internal equilibrium.	12.51570016
DIGESTION	Genes annotated by the GO term GO:0007586. The whole of the physical, chemical, and biochemical processes carried out by multicellular organisms to break down ingested nutrients into components that may be easily absorbed and directed into metabolism.	12.61618463
TRANSPORT	Genes annotated by the GO term GO:0006810. The directed movement of substances (such as macromolecules, small molecules, ions) into, out of, within or between cells.	14.49894074
REGULATION_OF_CELLULAR_PROCESCESS	Genes annotated by the GO term GO:0048522. Any process that activates or increases the frequency, rate or extent of cellular processes, those that are carried out at the cellular level, but are not necessarily restricted to a single cell. For example, cell communication occurs among more than one cell, but occurs at the cellular level.	15.0070049
ORGAN_DEVELOPMENT	Genes annotated by the GO term GO:0048513. Development of a tissue or	18.0867161

	tissues that work together to perform a specific function or functions. Development pertains to the process whose specific outcome is the progression of a structure over time, from its formation to the mature structure. Organs are commonly observed as visibly distinct structures, but may also exist as loosely associated clusters of cells that work together to perform a specific function or functions.	
SIGNAL_TRANSDUCTION	Genes annotated by the GO term GO:0007165. The cascade of processes by which a signal interacts with a receptor, causing a change in the level or activity of a second messenger or other downstream target, and ultimately effecting a change in the functioning of the cell.	36.99139983
**Wnt signaling pathway**		
	regulation	Log Fold change
KEGG_WNT_SIGNALING_PATHWAY		
WNT7B	up	12.54284933
SFRP2	up	7.631482266
FZD10	up	8.006648238
WNT4	up	4.587748424
DKK2	up	5.230394035
SERPINF1	up	3.66735327
SFRP4	up	4.572931405
WNT10A	up	4.043316091
CAMK2A	up	3.56755108
NKD2	up	2.638319087
NOTUM	up	5.031183408
WNT16	up	4.256767916
WNT3A	up	8.155359203

WNT11	up	2.213380123
FZD7	up	2.008235864
LRP5	up	1.918199863
RAC3	up	1.964931779
FZD1	up	1.561292217
CCND1	up	1.546855225
FOSL1	up	1.942984149
MYC	up	1.489094719
DAAM1	up	1.446087821
WNT3	up	6.679012085
SFRP1	up	1.406405548
DAAM2	up	1.492605457
VANGL2	up	1.601675161
WNT2	up	6.58048175
PPARD	up	1.265070934
PRKCG	up	6.360599613
FZD6	up	1.21116266
WNT10B	up	6.236663914
VANGL1	up	1.176110439
TCF7L1	up	1.252322924
**p53 signaling pathway**		
	regulation	Log Fold change
KEGG_P53_SIGNALING_PATHWAY		
GADD45A	up	1.6384392
CDK1	up	1.0706329
CCNB1	up	1.455245
CHEK1	up	1.0229402
SFN	up	1.2185273
THBS1	up	1.0243373
RRM2	up	1.4148407
SERPINB5	up	1.04601
**SHH signaling pathway**		
	regulation	Log Fold change
BIOCARTA_PTC1_PATHWAY		
CDC25B	up	1.0030532
CDK1	up	1.0706329
CCNB1	up	1.455245
CDC25A	up	1.1632915
**Tight junction**		
	regulation	Log Fold change
KEGG_TIGHT_JUNCTION		
RHOA	down	-1.329212
KRAS	down	-1.3890629

MYL9	up	1.4640789
MYH11	up	1.2676506
CLDN1	up	1.008378
JAM2	up	1.0531597
CLDN23	down	-1.7200146
CLDN8	up	1.5097151
CLDN2	down	-1.1935883
PARD3	down	-1.0856514
PPP2R2C	up	1.6813316
MPDZ	down	-1.1268911
**MAPK signaling pathway**		
	regulation	Log Fold change
KEGG_MAPK_SIGNALING_PATHWAY		
MAPK1	down	-1.005291
BRAF	down	-1.9218841
EGF	up	1.229434
PDGFRB	down	-1.0705147
KRAS	down	-1.3890629
FGF9	down	-1.2157922
FGF11	down	-1.248805
MAX	down	-1.5311418
FLNA	up	1.0267258
FLNC	up	1.1966758
CD14	up	1.2139683
PLA2G4A	down	-1.3123875
PLA2G4E	up	1.1018806
PLA2G1B	up	3.6469393
PPP3CC	up	1.0811634
HSPA1A	down	-2.2917461
HSPA1B	down	-1.4564962
HSPA8	down	-2.8714333
RASGRP3	up	1.1051178
MAP3K4	down	-1.0128765
DDIT3	down	-1.2584357
GADD45G	up	1.7765388
DUSP8	up	1.0669589
**Cell cycle**		
	regulation	Log Fold change
KEGG_CELL_CYCLE		
CDC25B	up	1.0030532
GADD45A	up	1.6384392
CDK1	up	1.0706329
CCNB1	up	1.455245

CDC25A	up	1.1632915
CHEK1	up	1.0229402
WEE1	down	-1.0280476
SFN	up	1.2185273
BUB1	up	1.3624682
CDC20	up	1.0923214
PTTG1	up	1.0892634
CCNA2	up	1.1491303
MCM3	up	1.3270435
MCM6	up	1.2899847
RBL1	up	1.376687
CDKN1C	down	-1.3907361
TTK	up	1.1134491
**Cytokine-cytokine receptor interaction**		
	regulation	Log Fold change
KEGG_CYTOKINE_CYTOKINE _RECEPTOR_INTERAERACTION		
EGF	down	-1.2542167
IL6	down	-1.2804132
CXCR4	up	2.41016
CXCL12	up	1.0495582
IL1B	down	-1.1590371
CCL5	up	1.128304
CCL2	down	-1.734571
CXCL1	down	-1.5471344
CXCL2	down	-1.8117905
CCL7	down	-1.3719487
IL18	down	-1.2492332
PRLR	down	-1.1920233
CXCL10	down	-2.2368584
CXCR6	up	1.259078
AMHR2	up	1.5519571
INHBA	up	1.9295492
BMPR1B	down	-1.64082
TNFSF13	up	1.4091592
CSF2RB	up	1.3097153
CLCF1	up	1.0542436
TNFSF12	up	1.4091592
TNFRSF9	up	1.1846724
TNFRSF11B	up	1.1218934
EDA2R	up	1.8851027
**Pathways in cancer**		
	regulation	Log Fold change

KEGG_PATHWAYS_IN_CANCER		
MAPK1	down	-1.005291
BRAF	down	-1.9218841
EGF	up	1.229434
PDGFRB	down	-1.0705147
PIK3R1	down	-1.0489173
PIK3CG	down	-1.4223032
BCL2	down	-1.363019
RHOA	down	-1.329212
FN1	up	1.0041542
LAMA3	down	-1.6419148
COL4A4	up	1.0572975
XIAP	down	-1.0693111
KRAS	down	-1.3890629
TCF7L2	down	-1.2056165
HSP90AA1	down	-1.4704523
FGF9	down	-1.2157922
FGF11	down	-1.248805
MAX	down	-1.5311418
CDKN2A	down	-1.1406264
RASSF5	down	-1.3410225
RARB	down	-1.0010996
PIAS2	down	-1.0214982
SKP2	down	-1.1205025
WNT5A	down	-1.5557861
WNT11	down	-1.0126076
FZD3	down	-1.1155128
CEBPA	down	-1.3592129
ZBTB16	up	1.3345199
RUNX1T1	up	1.3427367
IL6	up	1.059371
HHIP	down	-1.097496
**ECM-receptor interaction**		
	regulation	Log Fold change
KEGG_ECM_RECEPTOR_INTERACTION		
VWF	up	1.6774249
FN1	up	1.2487411
COL4A4	down	-1.6909618
LAMA5	up	1.1533756
ITGB5	up	1.0459785
SPP1	down	-2.1427765
THBS1	up	1.4211273
THBS4	down	-1.4994583

CHAD	up	1.3224831
COL1A1	up	1.2535119
COL6A1	up	1.0416222
COL6A3	up	1.1186771
COL11A2	up	1.7965012
TNC	up	1.5728073
IBSP	up	1.5691345
VTN	down	-1.8843503
CD36	down	-3.7551384
CD44	up	1.028532
**Focal adhesion**		
	regulation	Log Fold change
KEGG_FOCAL_ADHESION		
VWF	up	1.6774249
FN1	up	1.2487411
COL4A4	down	-1.6909618
LAMA5	up	1.1533756
ITGB5	up	1.0459785
SPP1	down	-2.1427765
THBS1	up	1.4211273
THBS4	down	-1.4994583
CHAD	up	1.3224831
COL1A1	up	1.2535119
COL6A1	up	1.0416222
COL6A3	up	1.1186771
COL11A2	up	1.7965012
TNC	up	1.5728073
IBSP	up	1.5691345
VTN	down	-1.8843503
EGF	down	-1.2542167
PIK3CG	up	1.0423455
PIK3R5	up	1.1262016
RAC2	up	1.1133113
PRKCA	down	-1.08985
MAPK1	up	1.0202107
CCND1	up	1.0458889
VAV1	up	1.2635775
ACTN1	up	1.3179817
CCND2	up	1.1588478
TLN1	up	1.2018456
ZYX	up	1.0275955
**Cell development**		
	regulation	Log Fold change

CELL_DEVELOPMENT		
GHRL	down	-1.3957701
EREG	down	-1.4710436
SCG2	down	-1.4179883
PTPRC	up	1.0943165
COL4A3	down	-1.0959458
TNFSF12	up	1.7902718
BLNK	up	1.5678139
COL4A4	down	-1.1126168
LRMP	up	1.9646859
GSTM3	down	-1.8490715
RTN1	down	-1.1720691
SFRP1	up	1.2786312
CD3G	up	1.3303871
F2	down	-1.4525471
SST	down	-1.8269787
BRCA1	up	1.0533907
IL6	up	1.1643963
CDKN2A	up	1.4537592
SFN	up	1.0424557
CXCR4	up	1.6672964
PDIA2	down	-1.7163515
IAPP	down	-2.016502
HSPA1B	up	1.5755863
SERPINB2	up	1.3293705
TIA1	down	-1.2858629
**Regulation of metabolic process**		
	regulation	Log Fold change
REGULATION_OF_METABOLIC_PROCESS		
TNFSF13	down	-1.2399406
EGF	up	1.229434
HTATIP2	up	1.4053993
BPTF	down	-1.0362315
FST	up	1.6560097
ZBTB16	up	1.3345199
PBX1	up	1.1598568
PBX4	up	1.0529194
GCLC	up	1.1652822
FLNA	up	1.0267258
TLE1	down	-1.1632271
RUNX2	down	-1.1863947
GHRL	up	1.9553108
SFTPD	down	-1.9200435

ARF6	down	-1.2989254
BRCA1	down	-1.2331982
NFATC3	down	-1.1582184
DDIT3	down	-1.2584357
CDKN2A	down	-1.1406264
MED13	down	-1.322217
ARID1A	down	-1.1020956
NR2F2	down	-1.4951596
TCF7L2	down	-1.2056165
RARB	down	-1.0010996
RBM14	down	-1.1570549
NAA16	down	-1.828979
UHRF1	down	-1.0276928
JMY	down	-1.2773342
GLIS3	down	-1.2624102
NRIP1	down	-1.1968746
SP1	down	-1.1812158
MED21	down	-1.6336899
CREB5	up	1.1255951
GABPB2	down	-1.4016094
DBP	up	1.2357368
ELF2	down	-1.0851135
MYCN	down	-1.3437319
KLF9	up	1.5134029
NFYA	down	-1.1325035
REST	down	-1.1134949
ITLN1	down	-3.283741
PIGA	down	-1.1560802
FURIN	up	1.7646408
RGMB	down	-1.684514
CDA	down	-1.2242432
HIVEP3	down	-1.3104672
PER1	up	1.1377678
CREBZF	down	-1.2673149
IL6	up	1.059371
CXCL12	up	1.1272974
HSP90AA1	down	-1.4704523
DNAJC1	down	-1.0882902
**Transcription**		
	regulation	Log Fold change
TRANSCRIPTION		
HTATIP2	up	1.4053993
BPTF	down	-1.0362315

FST	up	1.6560097
ZBTB16	up	1.3345199
PBX1	up	1.1598568
PBX4	up	1.0529194
GCLC	up	1.1652822
FLNA	up	1.0267258
TLE1	down	-1.1632271
RUNX2	down	-1.1863947
BRCA1	down	-1.2331982
NFATC3	down	-1.1582184
DDIT3	down	-1.2584357
CDKN2A	down	-1.1406264
MED13	down	-1.322217
ARID1A	down	-1.1020956
NR2F2	down	-1.4951596
TCF7L2	down	-1.2056165
RARB	down	-1.0010996
NR3C1	down	-1.1047964
RBM14	down	-1.1570549
NAA16	down	-1.828979
MTERF	down	-1.0190282
UHRF1	down	-1.0276928
JMY	down	-1.2773342
GLIS3	down	-1.2624102
NRIP1	down	-1.1968746
SP1	down	-1.1812158
MED21	down	-1.6336899
CREB5	up	1.1255951
GABPB2	down	-1.4016094
DBP	up	1.2357368
ELF2	down	-1.0851135
MYCN	down	-1.3437319
KLF9	up	1.5134029
NFYA	down	-1.1325035
REST	down	-1.1134949
POU2AF1	down	-1.4423838
CEBPA	down	-1.3592129
TARDBP	down	-1.0764599
MAX	down	-1.5311418
NFE2L2	up	1.0899525
TRIM24	down	-1.0719757
TSC22D1	down	-1.072535
FUBP1	down	-1.2215524

NFE2L3	down	-1.832304
GTF3C2	down	-1.5976171
RGMB	down	-1.684514
HIVEP3	down	-1.3104672
PER1	up	1.1377678
CREBZF	down	-1.2673149
**Regulation of cell proliferation**		
	regulation	Log Fold change
REGULATION_OF_CELL_PROLIFERATION		
GHRL	down	-1.3957701
EREG	down	-1.4710436
SCG2	down	-1.4179883
CXCL1	up	1.6614709
CXCL10	down	-1.546627
PTPRC	up	1.0943165
PTHLH	down	-3.7199984
COL4A3	down	-1.0959458
TNFSF12	up	1.7902718
PTN	down	-1.0660353
TNFSF13	up	1.7902718
SFTPD	up	2.6613064
COL18A1	up	1.3778
MATK	up	1.4839497
SST	down	-1.8269787
BRCA1	up	1.0533907
IL6	up	1.1643963
CDKN2A	up	1.4537592
ADAMTS1	down	-1.0817409
ADRA2A	up	1.4862471
TM4SF4	down	-1.1099949
QSOX1	up	1.0805035
UMOD	up	2.6298084
FABP3	down	-1.1761742
NDN	down	-1.232338
**Homeostatic process**		
	regulation	Log Fold change
HOMEOSTATIC_PROCESS		
GHRL	down	-1.6875305
CARTPT	down	-2.0665016
INHBA	up	1.9295492
CCL2	down	-1.734571
PLCE1	down	-1.3225574
S1PR3	down	-1.1846733

SFTPD	up	4.096679
CXCR4	up	2.41016
CCL5	up	1.128304
CXCL12	up	1.0495582
APOA1	down	-1.1174631
IL6	down	-1.2804132
CCKAR	down	-1.9879637
LPAR3	down	-1.4813807
EDNRA	up	1.3164873
BDKRB2	up	1.2384524
CCL7	down	-1.3719487
APOA4	down	-1.8381271
DDIT3	up	1.2419109
ABCG1	up	1.1083617
PPARGC1A	down	-1.056654
ATP7B	up	1.134037
SLC2A4	down	-1.2171054
TAC1	down	-1.6257362
ATP1A1	up	1.2417297
CP	up	1.1855607
**Digestion**		
	regulation	Log Fold change
DIGESTION		
SST	down	-1.9099722
SSTR2	down	-1.1303611
CCKAR	down	-3.639143
PPARGC1A	down	-1.0334282
PRSS2	down	-2.1736145
LDLR	up	1.1274023
APOA4	down	-1.559536
CEL	down	-2.1754665
PRSS3	down	-1.9937267
CTRL	down	-1.9721999
PGC	down	-2.8535042
CTSE	down	-1.8253536
TFF3	up	1.5173955
FABP2	up	1.3071041
ACSL1	down	-1.4626389
**Transport**		
	regulation	Log Fold change
TRANSPORT		
HTATIP2	up	1.4053993
KALRN	down	-1.3611517

FLNA	up	1.0267258
GHRL	up	1.9553108
CARTPT	up	1.1943421
ADORA1	up	1.0205803
AHSG	up	1.4043989
SFTPD	down	-1.9200435
CEP290	down	-1.4239969
AQP4	up	2.3561916
KCNMB2	up	1.0985298
ARF6	down	-1.2989254
SMG1	down	-1.4312344
NCBP2	down	-1.3031483
SGK1	up	1.6058846
WNK4	up	1.448504
ITLN1	down	-3.283741
SCG5	up	3.21772
LRP2	up	1.0952473
SLC28A2	down	-1.6004524
LMAN1	down	-1.009964
SLC44A2	down	-1.2155972
CD14	up	1.2139683
LYST	down	-1.0187321
HSP90AA1	down	-1.4704523
RIMS1	up	1.4222507
SLC7A6	down	-1.4182162
GJA4	up	1.0118132
CKLF	down	-1.4977131
BCL2	down	-1.363019
SLC2A4	up	1.4460735
CAV1	up	1.3255925
KIF1B	down	-2.2199187
SNAP25	up	2.2673836
SLC7A8	up	1.7466493
SLC7A5	up	1.08149
NNT	up	1.1606407
SCNN1G	up	1.1231885
AQP5	down	-1.4068298
KCNQ1	up	1.117199
KCNE2	up	1.5570717
FXYD1	up	1.2804737
CHKA	down	-1.0385981
RAB14	down	-1.4923482
SLC25A4	up	1.2631683

KIF13B	down	-1.1090236
PLN	up	2.3711448
VTI1A	down	-1.9212847
TNPO1	up	1.4191127
KDELR1	down	-1.0202694
ITSN1	down	-1.0520501
SLC2A3	up	1.140049
SLC35B4	down	-1.0941014
SLC38A3	up	1.2071767
TOMM20	up	1.1075397
SLC18A2	up	2.0950584
KCND3	up	1.0377064
PLP2	up	1.4755135
SLC16A7	up	1.2703743
SLCO2A1	up	1.2876945
GC	up	2.8358116
CLCA1	down	-1.512907
CLCA2	down	-2.3475418
**Regulation of cellular processess**		
	regulation	Log Fold change
REGULATION_OF_CELLULAR_PROCESCESS		
TNFSF13	down	-1.2399406
EGF	up	1.229434
HTATIP2	up	1.4053993
BPTF	down	-1.0362315
MATK	down	-1.0927753
BMPR1B	up	1.277329
PTHLH	up	2.4065495
FLNA	up	1.0267258
GHRL	up	1.9553108
SCG2	up	2.0676136
CXCL10	up	3.194405
CARTPT	up	1.1943421
ADORA1	up	1.0205803
AHSG	up	1.4043989
TNFSF12	down	-1.2399406
PTN	up	1.2225266
SFTPD	down	-1.9200435
BTG1	up	1.2187767
COL4A4	up	1.0572975
S1PR3	up	1.6159883
ARF6	down	-1.2989254
BRCA1	down	-1.2331982

CDKN2A	down	-1.1406264
MED13	down	-1.322217
ARID1A	down	-1.1020956
RBM14	down	-1.1570549
NAA16	down	-1.828979
NCBP2	down	-1.3031483
JMY	down	-1.2773342
GLIS3	down	-1.2624102
NRIP1	down	-1.1968746
SP1	down	-1.1812158
MED21	down	-1.6336899
CREB5	up	1.1255951
ITLN1	down	-3.283741
RGMB	down	-1.684514
HIVEP3	down	-1.3104672
IL6	up	1.059371
CDKN2AIP	down	-1.4816163
VIP	up	1.1766887
SST	up	1.7829819
IL15	down	-1.1403208
ADRA2A	down	-1.8173199
SLC44A2	down	-1.2155972
MAPK1	down	-1.005291
LTA	up	1.0479474
RHOA	down	-1.329212
EEF1D	up	1.3376822
GOLT1B	down	-1.0097885
TSPAN6	down	-1.4138222
HSP90AA1	down	-1.4704523
NME1	down	-1.1151924
TBC1D8	up	1.0291815
LRP5	down	-1.0415449
CDC7	down	-1.0696831
CAPN1	down	-1.022141
IKBIP	down	-1.4137001
TIA1	down	-1.2629023
**Organ development**		
	regulation	Log Fold change
ORGAN_DEVELOPMENT		
GHRL	down	-1.6875305
CARTPT	down	-2.0665016
INHBA	up	1.9295492
CCL2	down	-1.734571

PLCE1	down	-1.3225574
EREG	up	2.4327269
ANG	down	-1.6477761
CXCL10	down	-2.2368584
SCG2	down	-2.2867355
BLNK	up	1.2079773
SHH	down	-1.6509748
RET	down	-1.3430524
PTHLH	down	-3.457803
EGF	down	-1.2542167
COL4A3	down	-1.4173126
IGFBP4	up	1.3967228
TNFSF12	up	1.4091592
FGFR1	up	1.0256958
TNFRSF11B	up	1.1218934
SFTPD	up	4.096679
COL4A4	down	-1.6909618
KRT1	down	-1.1246834
CTGF	up	1.964344
DHCR24	down	-1.1365013
IL18	down	-1.2492332
MATK	up	1.5888643
BMPR1B	down	-1.64082
BMX	up	1.8797255
ASPRV1	down	-1.0213175
GPR56	up	1.1561298
ZBTB16	down	-1.3576946
PAX6	down	-1.0830364
PHGDH	down	-1.6949673
COL18A1	up	1.6947308
TNNI3	down	-1.9687138
LRMP	up	1.2332554
PTF1A	down	-1.0280457
HAND2	down	-1.0026498
EGFL7	up	1.0411377
PDX1	down	-1.2492223
TBX4	up	1.6804466
FABP1	down	-2.1919708
ITGAX	up	1.3456688
ONECUT2	up	1.6777687
DMP1	up	1.600709
SPARC	up	1.177166
COL1A1	up	1.2535119

KRT10	down	-1.4294915
PAPSS2	down	-1.734684
EGR3	up	2.0333805
VAMP5	up	1.5124512
COL6A3	up	1.1186771
**Signal transduction**		
	regulation	Log Fold change
SIGNAL_TRANSDUCTION		
GHRL	down	-1.6875305
CARTPT	down	-2.0665016
INHBA	up	1.9295492
CCL2	down	-1.734571
PLCE1	down	-1.3225574
EREG	up	2.4327269
ANG	down	-1.6477761
CXCL10	down	-2.2368584
CXCL1	down	-1.5471344
SCG2	down	-2.2867355
BLNK	up	1.2079773
SHH	down	-1.6509748
RET	down	-1.3430524
PTHLH	down	-3.457803
EGF	down	-1.2542167
PTN	down	-1.2294049
COL4A3	down	-1.4173126
IGFBP4	up	1.3967228
TNFSF12	up	1.4091592
FGFR1	up	1.0256958
TNFRSF11B	up	1.1218934
FGF13	down	-1.1368122
VLDLR	down	-1.5140953
TNFSF13	up	1.4091592
S1PR3	down	-1.1846733
ARF6	up	1.1249518
CAP2	down	-1.1290092
CD3G	up	1.5484052
WNT5A	up	2.122489
F2	down	-1.444807
PRLR	down	-1.1920233
EDN3	down	-1.1537871
CXCR4	up	2.41016
CCL5	up	1.128304
CXCL12	up	1.0495582

APOA1	down	-1.1174631
IL6	down	-1.2804132
CCKAR	down	-1.9879637
LPAR3	down	-1.4813807
VIP	down	-1.7933316
EDNRA	up	1.3164873
BDKRB2	up	1.2384524
CCL7	down	-1.3719487
CDKN2A	up	3.8307738
ANXA1	up	1.1919727
PDIA2	down	-1.2505379
FPR2	down	-1.8694401
SGK2	down	-1.2716522
ATRIP	down	-1.3825989
PTPRD	down	-1.0818024
FMOD	down	-1.6343575
CLCF1	up	1.0542436
DUSP4	up	1.2367401
MAPK6	up	1.0455956
CD74	up	1.1625347
SST	down	-2.4912539
SSTR2	down	-1.1473513
FPR1	down	-1.352808
MIF	up	1.0907335
HPGD	down	-2.1417165
MPZL1	up	1.2085629
TACR2	down	-1.1014061
PTGER3	down	-1.5076504
ADRA2A	up	1.95994
MS4A2	up	1.0615563
GCG	down	-1.1644115
RGS1	up	1.3302732
P2RY1	up	1.182436
CXCR6	up	1.259078
GNG4	down	-1.2650166
GRP	down	-1.5998483
RAPGEF4	down	-2.6853104
GABRA1	down	-2.5931952
GABRA4	down	-1.7648916
RASD1	down	-1.1589365
ENPP2	down	-1.09128
PIK3CG	up	1.0423455
C3	up	1.3172159

LGR5	down	-1.2434406
CDKN1C	down	-1.0875721
SNX6	down	-1.091959
LTBP2	up	1.1919727
TACSTD2	up	1.077672
HIPK2	down	-1.2035484
RGMB	down	-1.0054207
GEM	down	-1.0004673
GFRA2	down	-1.0587344
LY6E	up	1.0580482
MAPK1	up	1.0202107
NR1H4	down	-1.0670247
IL1B	down	-1.1590371
KCNIP1	down	-1.7109385
IAPP	down	-2.9282503
ZYX	up	1.0275955
WISP1	up	1.4310784
SOCS3	down	-1.3403645
CFL1	up	1.1356964
AMBP	down	-1.971066
CLEC7A	up	1.6958799
SLC20A1	up	1.7731209
RARB	up	1.167366
TLR2	down	-1.2424164
EDA2R	up	1.8851027
PLK2	up	1.5063858
TSPAN6	up	1.6859722
DDAH1	up	1.1013603
GZMB	up	1.1005006
CARD10	up	1.2855053
PLCL1	down	-1.0192671
PLA2G1B	down	-4.2658386
AMHR2	up	1.5519571
ANGPT2	up	1.1505585
NR4A1	up	2.6414857
NR3C2	down	-1.6260433
P2RX7	up	1.0191646
PENK	down	-1.7643852
CYTL1	down	-1.3379211
PPIC	up	1.4392815
RAC2	up	1.1133113
IRS4	down	-1.6310201
IQGAP1	up	1.0744762

PSTPIP1	down	-1.2270856
IL1RL1	up	1.1704712
RIN1	up	1.120367
