# Supplementary material for: SLC26A9 deficiency causes gastric intraepithelial neoplasia in mice and aggressive gastric cancer in humans
Source: Cell Oncol (Dordr). 2022 Apr 14;45(3):381–98. doi: 10.1007/s13402-022-00672-x (PMC9187568; doi:10.1007/s13402-022-00672-x)

Figure 5C

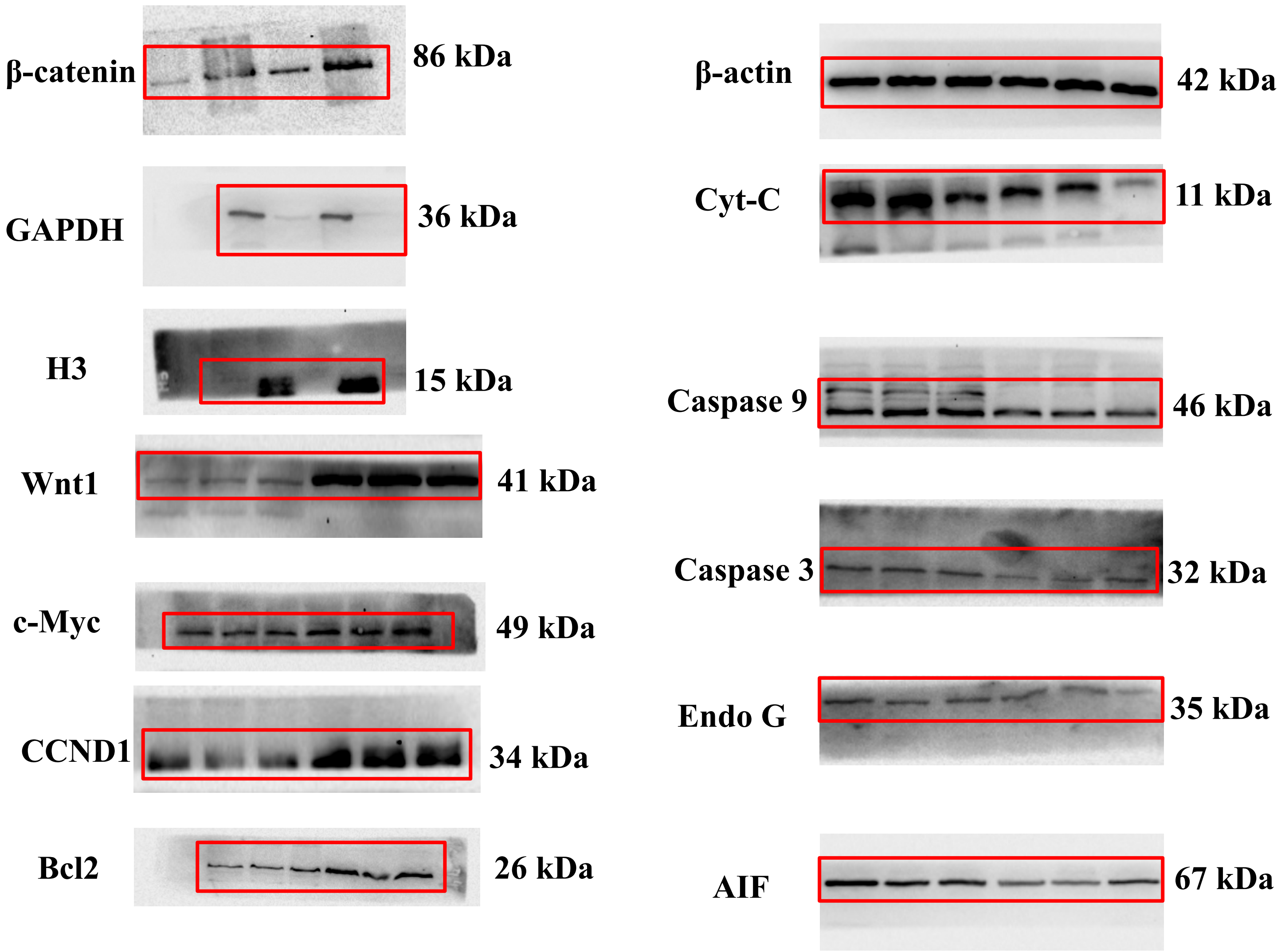
β-catenin
86 kDa
GAPDH
36 kDa
H3
15 kDa
Wnt1
41 kDa
c-Myc
49 kDa
CCND1
34 kDa
Bcl2
26 kDa
β-actin
42 kDa
Cyt-C
11 kDa
Caspase 9
46 kDa
Caspase 3
32 kDa
Endo G
35 kDa
AIF
67 kDa

Figure 7F

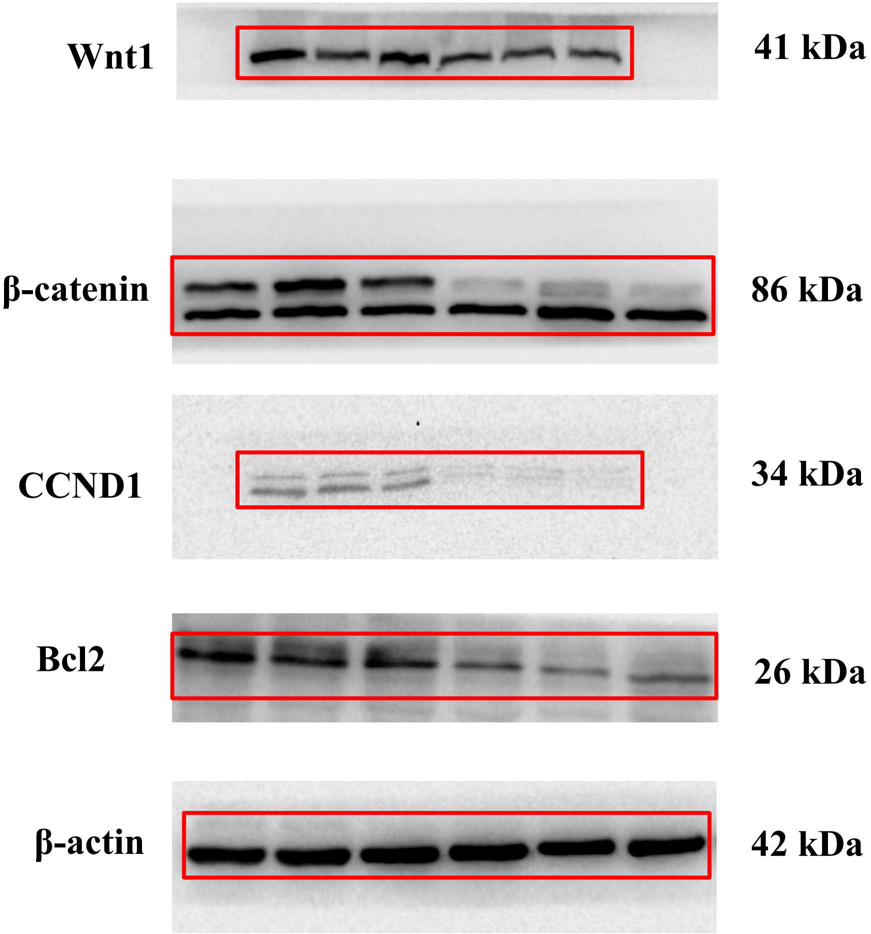
Wnt1
41 kDa
β-catenin
86 kDa
CCND1
34 kDa
Bcl2
26 kDa
β-actin
42 kDa

Supplementary Figure 1B

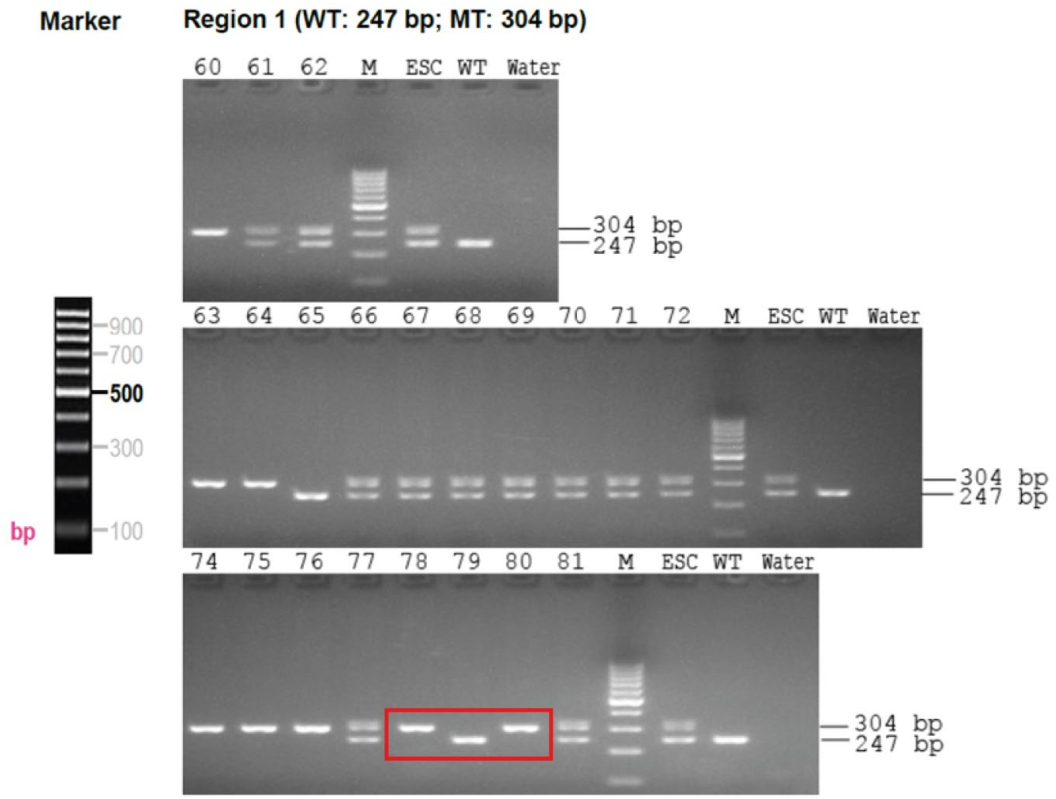

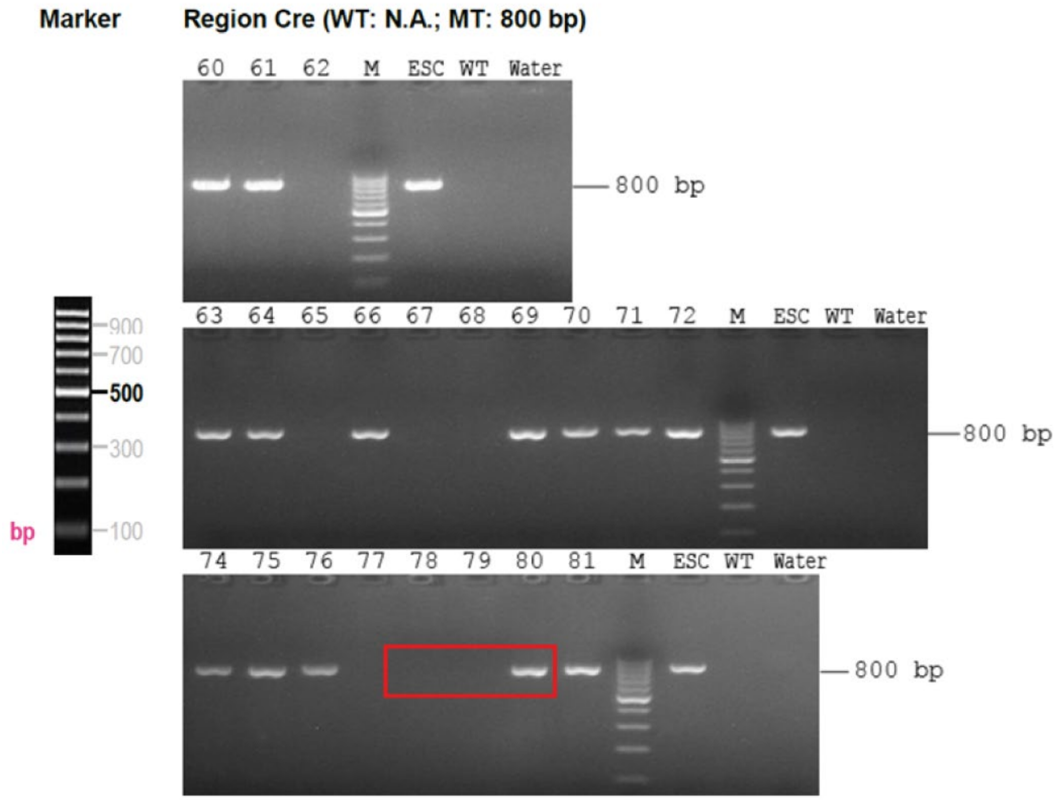

Supplementary Figure 1C

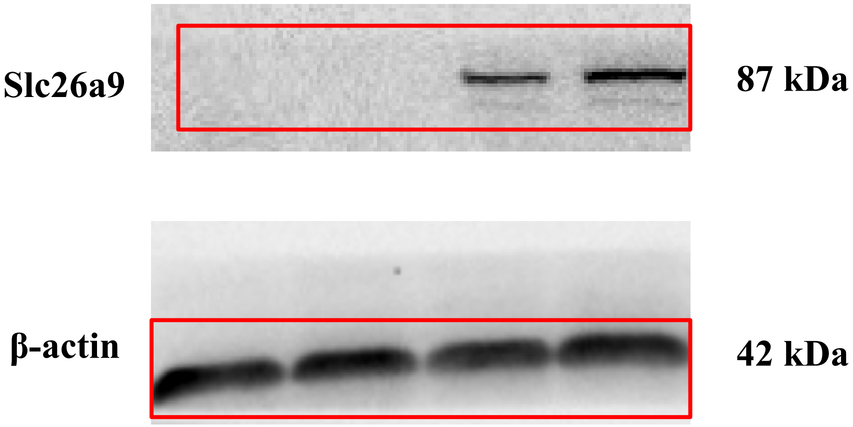

Supplementary Figure 3A

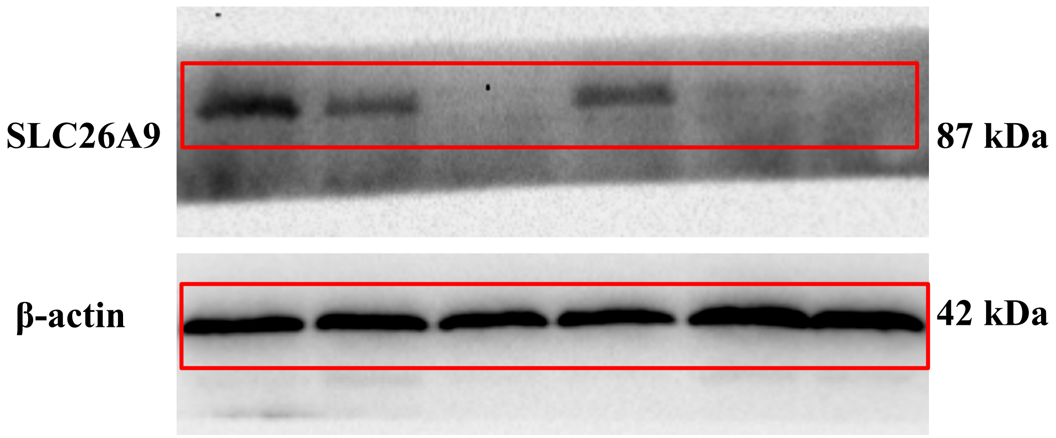

Supplementary Figure 3B

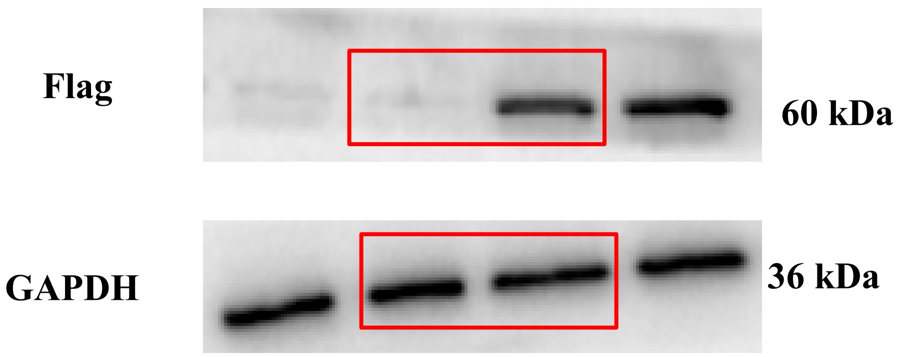

Supplement: Supplementary file 7 — Supplementary file7 (PDF 1323 KB) [file 13402_2022_672_MOESM7_ESM.pdf]
